# Comparative efficacy of various CHIs combined with western medicine for non-small cell lung cancer: A bayesian network meta-analysis of randomized controlled trials

**DOI:** 10.3389/fphar.2022.1037620

**Published:** 2022-11-10

**Authors:** Ciyan Peng, Jing Chen, Wei Cui, Sini Li, Jianhe Li, Liubao Peng

**Affiliations:** ^1^ Department of Pharmacy, The Second Xiangya Hospital of Central South University, Changsha, China; ^2^ Department of Pharmacy, The First Affiliated Hospital of Hunan University of Chinese Medicine, Changsha, China

**Keywords:** network meta-analysis, bayesian model, Chinese herbal injections, non-small cell lung cancer, combined therapy, Chinese medicine

## Abstract

**Background**: Given the limitations of Western medicine (WM) for the treatment of non-small cell lung cancer (NSCLC) and the wide exploration of Chinese herbal injections (CHIs), systematically evaluate the efficacy of Various CHIs Combined with WM for Non-small Cell Lung Cancer. In this study, we performed a network meta-analysis to evaluate the comparative efficacy of 16 CHIs combined with WM regimens for the treatment of NSCLC.

**Methods**: Literature databases were searched from their inception to November 2021, and all randomized control trials (RCTs) involving NSCLC patients treated with a combination of Chinese and WM were retrieved. Outcomes, including disease control rate, survival quality score, incidence of gastrointestinal adverse reactions, incidence of leukopenia, and incidence of thrombocytopenia, were analyzed using RevMan (5.3), Stata17, and R software. Surface under the cumulative ranking curve (SUCRA) probability values were calculated to rank the treatments examined, and clustering analysis was used to compare the effects of CHIs on different outcomes.

**Results**: A total of 389 studies involving 31,263 patients and 16 CHIs were included. The 16 CHIs were: Aidi injection (ADI), Huachansu injection (HCSI), oil of Ophiopogon injection (OOMI), disodium cantharidinate and vitamin B6 injection (DCI), Shenfu injection (SFI), Shenmai injection (SMI), Shenqi Fuzheng injection (SQFZI), Chansu injection (CSI), Delisheng injection (DLSI), Fufang Kushen injection (FFKSI), Huangqi injection (HQI), Kangai injection (KAI), Kanglaite injection (KLTI), Shengmai injection (SI), Xiangguduotang injection (XGDTI), and Xiaoaiping injection (XAPI). The results of the network meta-analysis showed that, with WM treatment as a co-intervention, CSI was most likely to improve the disease control rate (SUCRA = 80.90%), HQI had the highest probability of being the best option for improving the survival quality score (SUCRA = 82.60%), DCI had the highest probability of reducing the incidence of gastrointestinal adverse reactions (SUCRA = 85.50%), HCSI + WM had the highest probability of reducing the incidence of thrombocytopenia (SUCRA = 91.30%), while SMI had the highest probability of reducing the incidence of leukopenia (SUCRA = 79.10%).

**Conclusion**: CHIs combined with WM is proved to be more effective than WM alone, which may be beneficial to NSCLC patients. SMI + WM and DCI + WM are most likely the optimal CHI to improve disease control rates, survival quality score, and reduce adverse effects. This study has limitations; therefore, higher quality RCTs and real-world evidence are required to support our conclusions.

## 1 Introduction

Lung cancer is the malignancy with the highest mortality and incidence rates, both worldwide and in China (Bray et al., 2018; Sung et al., 2021; Siegel et al., 2021; Alexander et al., 2020). Lung cancer brings a tremendous economic and social burden on both developing and developed countries (Minguet et al., 2016; Sung et al., 2021; Bray et al., 2018; Molina et al., 2008). Based on histology, Lung cancer can be classified as non-small cell lung cancer (NSCLC) and small cell lung cancer. Non-small cell lung cancer (NSCLC) accounts for approximately 80%–85% of all lung cancers (Miller et al., 2020; Miller et al., 2016; Miller et al., 2022).

Currently, platinum based chemotherapy is still the first-line treatment for lung cancer (Ettinger et al., 2017; Ettinger et al., 2022). However, some patients are unable to complete the recommended cycles of chemotherapy due to serious adverse events, greatly limiting its clinical application (Quoix et al., 2011; Shojaee and Nana-sinkam, 2017; Ettinger et al., 2022). With the development of modern treatment methods and biotechnology, lung cancer has entered the era of precision therapy (Soo et al., 2017; Hirsch et al., 2017; Yoneda et al., 2018). Molecular targeted drugs and immunotherapy have substantially improved the clinical efficacy of mid-to late-stage lung cancer treatment (Lemjabbar-Alaoui et al., 2015; Cho, 2017; Zhang et al., 2018; Jiang et al., 2019; Li et al., 2021). Although Immunotherapy increasing the 5-year survival rate of patients with advanced NSCLC from 5% to approximately 23% (Reck et al., 2022; Ren et al., 2021; Yoneda et al., 2018; Garon et al., 2019; Breimer et al., 2020), it is often accompanied by low antigenicity, side effects, and drug resistance for lung cancer has limitations in clinical practice (Khunger et al., 2018; Zhang et al., 2018; Xia et al., 2019; Wang et al., 2021; Stock-Martineau et al., 2021; Zhou and Zhou., 2021). Therefore, how to actively identify effective drug treatment options to reduce postoperative recurrence and metastasis, prolong the survival time of patients with advanced disease, reduce adverse effects, and improve patient quality of life remains a key challenge in the treatment of advanced lung cancer.

Chinese medicine injections (CHIs) are widely used by clinicians in China as an important component of complementary and alternative medicine for the adjuvant treatment of NSCLC (Jiao et al., 2017; Jiang et al., 2019; Xu et al., 2021; Han et al., 2016; Wu et al., 2016; He et al., 2016; Cao et al., 2017; Cao et al., 2021; Wang B et al., 2018). Many studies have documented the efficacy of Various CHIsCombined with Western Medicine for Non-small Cell Lung Cancer (Li et al., 2021; Zhang et al., 2018; Yao et al., 2020; Jiang et al., 2016; Jiang et al., 2019; Tan and Wang., 2016; Liu et al., 2017; Ma and Xu., 2017; Zheng et al., 2017; Wang G et al., 2018; Li et al., 2020; Liu et al., 2021; Xie et al., 2021). However, there are various types of CHIs, and the optimal strategy for combining CHIs with WM for treating NSCLC remains inconclusive, which may cause difficulty for clinicians in clinical treatment. Bayesian network meta-analysis (NMA) has the advantage of combining direct and indirect evidence to compare multiple interventions (Schwingshackl et al., 2019; Watt et al., 2019; Ahn and Kang., 2021; González-Xuriguera et al., 2021; Watt and Del Giovne, 2022). Therefore, in this study, we used Bayesian network meta-analysis (NMA) method to systematically evaluate the efficacy of Various CHIs Combined with WM for Non-small Cell Lung Cancer. The objective of this NMA was to supplement the optimal strategy of NSCLC treatment and to strengthen additional insights for clinical practice in the future.

## 2 Methods

This study is reported in strict accordance with the standard format specifications detailed in the Preferred Reporting Items for Systematic Reviews and Meta-Analysis: PRISMA Extension Statement (Cornell, 2015; Hutton et al., 2015; Rethlefsen et al., 2021). A completed PRISMA checklist is included as an additional file (Supplementary File). The study protocol was registered and approved on the International Platform of Registered Systematic Review and Meta-Analysis Protocols (INPLASY) on 17 November 2021 (registration number: INPLASY2021110068), and the study was conducted strictly in accordance with the registered study protocol. This NMA did not require ethical approval, because the study only collected clinical data from each randomized controlled trial (RCT) and did not disclose patient information.

### 2.1 Search strategy

In this NMA, a comprehensive data search of the following electronic databases for RCTs of CHIs combined with WM for NSCLC was conducted from their inception to November 2021: Chinese Biological Medicine Literature, China National Knowledge Infrastructure, Wanfang, PubMed, Cochrane Library, and Embase. In addition, pharmaceutical companies that manufacture proprietary CMI were contacted to provide unpublished information regarding premarket and postmarket studies. Further, study authors were contacted to supplement incomplete reports of original papers or to provide data from unpublished studies. The search strategy was divided into three parts: CHIs, NSCLC, and RCTs. A search strategy was developed, as illustrated in Table 1 using PubMed as an example. A total of 16 CHIs that met the national standards of the Chinese Food and Drug Administration were included (https://db.yaozh.com and https://www.nmpa.gov.cn/). Detailed drug information is provided in (Table 1 and Supplementary Table S1).

**TABLE 1:** Specific terms used to search PubMed.

#1 Non-small cell lung cancer (MeSH terms), #2 carcinoma, Non-small cell lung (title/Abstract), #3 carcinomas, Non-small-cell lung (title/Abstract), #4 lung carcinoma, Non-small-cell (title/Abstract), #5 lung carcinomas, Non-small-cell (title/Abstract), #6 Non-small-cell lung carcinoma (title/Abstract), #7 Non-small-cell lung carcinomas (title/Abstract)
#8, #1 OR #2 OR #3 OR #4 OR #5 OR #6 OR #7
#9 Aidi Injection (Title/Abstract), #10 Huachansu Injection (Title/Abstract), #11 Oil of Ophiopogon Injection (Title/Abstract), #12 Disodium Cantharidinate and Vitamin B6 Injection (Title/Abstract), #13 Shenfu Injection (Title/Abstract), #14 Shenmai Injection (Title/Abstract)
#15 Shenqifuzheng Injection (Title/Abstract), #16 Chansu Injection (Title/Abstract), #17 Delisheng Injection (Title/Abstract), #18 Fufangkushen Injection (Title/Abstract), #19 Huangqi Injection (Title/Abstract)
#20 Kangai Injection (Title/Abstract), #21 Kanglete Injection (Title/Abstract), #22 Shengmai (Title/Abstract), #23 Xiangguduotang Injection (Title/Abstract), #24 Xiaoaiping Injection (Title/Abstract)
#25 #9 OR #10 OR #11 OR #12 OR #13 OR #14 OR #15 OR #16 OR #17 OR #18 OR #19 OR #20 OR #21 OR #22 OR #23, OR #24
#26 randomized controlled trial (Publication type)
#27 controlled clinical trial (Publication type)
#28 #26 OR # 27
#28 #8 AND #25 AND #28

### 2.2 Inclusion criteria

#### 2.2.1 Study types

RCTs that reported the efficacy of the 16 CHIs combined with WM for treating NSCLC were eligible. There were no language restrictions. When outcome information was available for multiple time points, the data for the longest follow-up time point were selected.

#### 2.2.2 Participants

All patients were pathologically and histologically diagnosed with NSCLC. There were no limitations on sex, age, race, region or nationality.

#### 2.2.3 Interventions

Patients in control groups received only WM regimens, including DP, TP, GP, NP, and GE, where D indicates docetaxel, P cisplatin, T paclitaxel, G gemcitabine, N vinorelbine, and E gefitinib. Patients in the treatment group received CHIs together with WM therapy. To facilitate our analyses, we collapsed all agents regardless of dose. If patients had complications during the treatment, then some appropriate mitigation measures could be taken.

### 2.3 Outcome measures

The primary outcome indicator was DCR, where DCR was defined as complete remission + partial remission + stable. Survival quality was assessed using the Karnofsky performance scale (KPS), with improvement defined as an increase of ≥10 points in the KPS score after treatment, stability as an increase or decrease of <10 points, and a decline as a decrease of ≥10 points in the KPS score. Secondary outcome indicators were the incidence of adverse reactions, including gastrointestinal (GI) adverse reactions, leukopenia, and thrombocytopenia. Data were extracted according to the predefined definitions described in the protocol, with priority given to the earliest published report when data appeared in more than one report. RCTs were eligible if they reported one of the aforementioned outcomes.

### 2.4 Exclusion criteria

The exclusion criteria were as follows: 1) Other western medical treatment options (Radiotherapy, Surgical and Local interventional therapy); 2) Other traditional Chinese Medicine treatment options (Decoction for oral and external use, emotional therapy, static breathing control, acupuncture); 3) repeat publications; and 4) Studies with incomplete or incorrect data.

### 2.5 Data extraction and quality assessment

All retrieved studies were managed using EndNote software. After excluding duplicate studies, two researchers (Jing Chen and Sini Li) independently screened the retrieved studies according to the inclusion and exclusion criteria, and after excluding unrelated literature, such as reviews, evaluations, animal studies, and uncontrolled studies, the full text of each report was read to finalize the texts for inclusion in the study and to extract the data. Subsequently, a review team consisting of two researchers (Jianhe Li and Wei Cui) checked the accuracy of the data and assessed the quality of the included studies. Information extracted from included studies comprised: study name, study date, number of patients, sex ratio, treatment strategy, treatment procedure, and outcomes.

Two researchers (Jianhe Li and Wei Cui) independently assessed the risk of bias in included RCTs according to the risk of bias tool provided in the Cochrane Handbook for Systematic Reviews of Interventions. The following were assessed: 1) selection bias associated with random sequence generation; 2) selection bias associated with allocation concealment; 3) performance bias: blinding of participants and personnel; 4) detection bias: blinding of outcome assessments; 5) attrition bias: completeness of outcome data; 6) reporting bias: selective reporting; and 7) other sources of bias. Each factor was categorized as “low risk”, “high risk”, or “unclear”. All discrepancies that emerged from this study were discussed by a review panel.

### 2.6 Data analysis

All meta-analyses were performed within a Bayesian framework using R4.11 software for statistical analysis of data and research, and a Markov chain Monte Carlo method for Bayesian inference. The parameters set in R4.11 software were as follows: number of chains, 4; tuning iterations, 50,000; simulation iterations, 100,000; thinning interval, 1; settings of tuning iterations and simulation iterations were adjusted according to the actual situation. Potential scale reduction factors were used to evaluate the convergence of Markov chains. Models were compared using the deviance information criterion, which is equal to the sum of the posterior mean of the residual deviations and the number of valid parameters. Results for comparisons of dichotomous variables were calculated as odds ratios (OR). Differences between groups were considered statistically significant when the 95% confidence interval (CI) of OR values did not contain 1. Network diagrams showing indirect comparative relationships among different interventions were generated, where the nodal areas for each intervention represent the number of patients, and the thickness of lines between different interventions represented the number of RCTs. R4.11 and Stata17 software were used to plot cumulative probability ranking, and to generate mesh and funnel plots for each intervention. A surface under the cumulative ranking area (SUCRA) curve is used to estimate the probability of ranking each intervention; the larger the area under the curve, the higher the ranking and the higher the probability that the CHIs are the best interventions (Salanti et al., 2011). Clustering analysis was used to synthesize and compare interventions with two different outcome indicators, to obtain the best choice of injection for both outcome indicators: the farther away from the origin in the clustering plot, the better the outcome indicator. Acomparison-adjusted funnel plot was used to assess potential publication bias. If points on both sides of the midline in the funnel diagram were symmetric, which meant the correction guideline was at right angles to the midline, it was considered indicative of no significant publication bias.

## 3 Results

### 3.1 Search results

**FIGURE 1:**
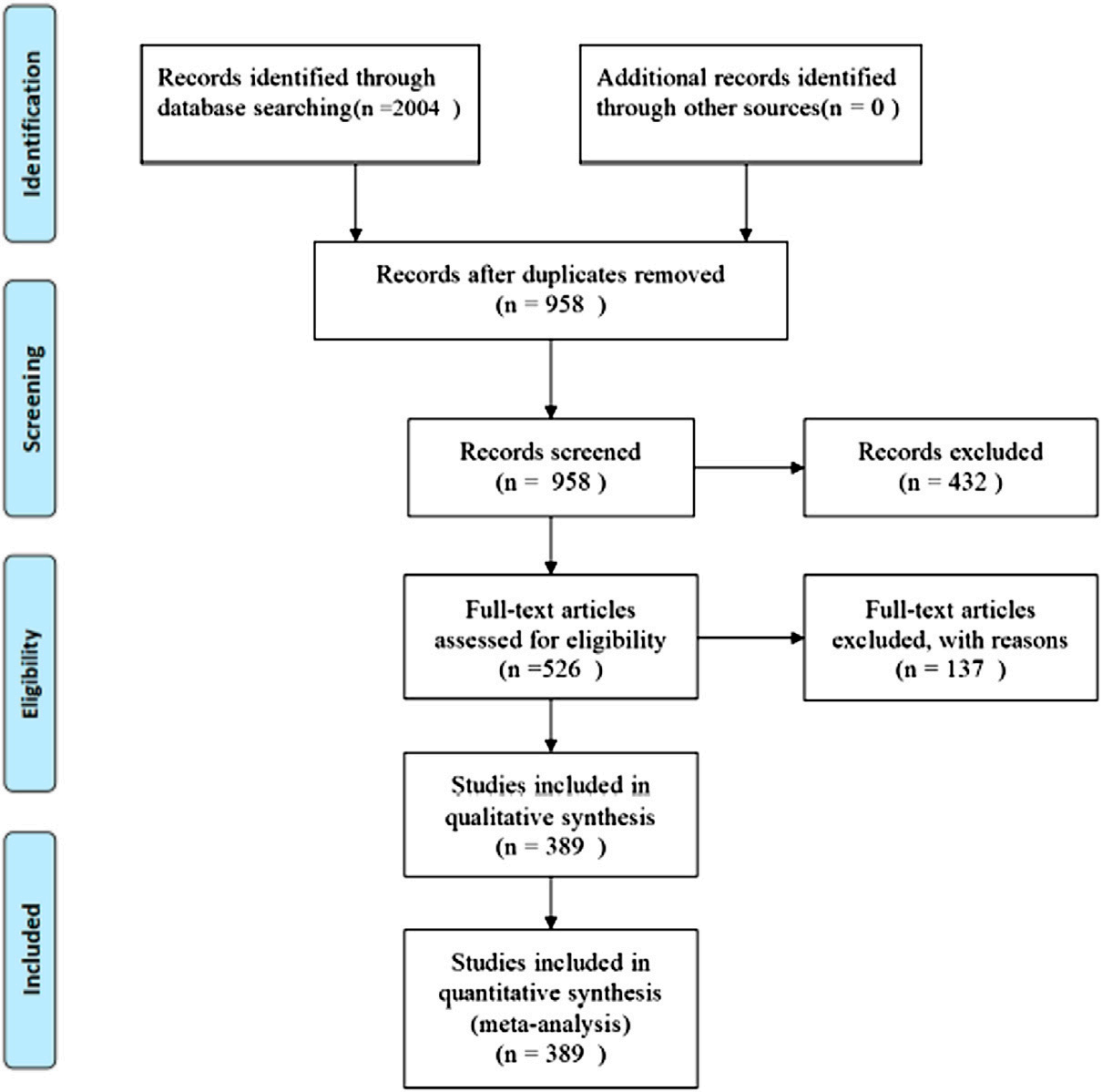
PRISMA flow diagram.

A total of 2004 studies were retrieved, and 389 RCTs (Supplementary Table S2) were finally included for NMA according to the pre-defined inclusion and exclusion criteria. Further details of the literature screening process are shown in Figure 1. The 389 RCTs reported 16 herbal injections used as interventions in combination with conventional WM, as follows: Aidi injection (ADI, 66 RCTs), Huachansu injection (HCSI, 10 RCTs), oil of *Ophiopogon* injection (OOMI, 23 RCTs), disodium cantharidinate and vitamin B6 injection (DCI, 14 RCTs), Shenfu injection (SFI, 14 RCTs), Shenmai injection (SMI, 21 RCTs), Shenqi Fuzheng injection (SQFZI, 39 RCTs), Chansu injection (CSI, 4 RCTs), Delisheng injection (DLSI, 8 RCTs), Fufang Kushen injection (FFKSI, 55 RCTs), Huangqi injection (HQI, 8 RCTs), Kangai injection (KAI, 37 RCTs), Kanglaite injection (KLTI, 39 RCTs), Shengmai injection (SI, 11 RCTs), Xiangguduotang injection (XGDTI, 18 RCTs), and Xiaoaiping injection (XAPI, 22 RCTs) (Table 2). Of the 389 RCTs reported, 306 reported DCR, 198 reported Survival Quality Score, 222 reported Incidence of GI Adverse Reactions, 198 reported Incidence of Leukopenia, and 113 reported Incidence of Thrombocytopenia. All included studies were published in Chinese, and the publication years were from 2003 to 2021.

**TABLE 2:** Detailed information on Chinese herbal injections.

Chinese herbal injection (Name of the formulation)	Name of the herbal drug	Functional indications	Number of articles	Number of patients	
				Number of men	Number of women
ADI	Cantharis, ginseng, astragalus, acanthopanax senticosus	anti-tumor and immunomodulatory effects Cichello et al. (2015); Meng et al. (2018); Ge et al., 2017; Wang et al. (2018)	66	3,681	2,339
XAPI	Tongguanteng extract, polysorbate	target apoptosis and autophagy leading to the death of NSCLC cells death Jiao et al. (2018)	22	956	618
XGDTI	Lentinan	Enhance the immunity of the body and enhance thesusceptibility Xu et al. (2020); Lv et al. (2021)	18	898	497
CSI	Toad	Induce apoptosis, promote cell differentiation, increase immunity, inhibit vascular proliferation and endothelial cell proliferation Su and Niu (2001); Efferth et al. (2002)	4	114	153
DCI	Sodium cantharidate, vitamin B6	Inhibit the synthesis of protein and nucleic acid in tumor cells and reduce the level of cancer toxin Wang et al. (2019)	14	591	390
DLSI	Red Ginseng, *Astragalus*, Toad, Cantharis	Enhance the inhibition of chemotherapy on tumor metastasis Dong et al. (2014)	8	378	184
FFKSI	*Sophora* flavescens, Smilax glabra	Enhance the resistance of body cancer cells, promote the apoptosis of cancer cells, reduce the stimulation to the body, Anti inflammation, endocrine regulation and immune function improvement Zhang et al. (2013); Agrawal et al. (2014)	55	2,933	1715
HCSI	Dry Toad Skin Extract	inhibit tumor cells, reduced the ADRs in patients with advanced NSCLC Tan et al. (2021)	10	577	412
HQI	*Astragalus* membranaceus	Inhibiting tumor cell proliferation, affecting tumor tissue metabolism and Functions such as regulating immunity Sang et al. (2008)	8	331	156
KAI	*Astragalus* membranaceus, ginseng, matrine	matrine inhibits the proliferation and metastasis of tumor cells by inducing apoptosis, halting the cell cycle, and inhibiting the formation of blood vessels; *Astragalus* membranaceus induction of interferon or exert interferon-like effects, and to enhance anti-tumor effects by strengthening the activity of NK cells Zhao et al. (2012); Li, T et al. (2019); Hu et al. (2022)	37	1835	1,063
KLTI	Coix seed oil, soybean phospholipid	induction of cancer cell apoptosis, inhibition of cancer cell mitosis, execution of cancer cells, and improvement of the immune function Gui and Dai, (2020)	39	2,101	1,417
OOMI	Refined Brucea javanica oil, refined soybean phospholipid, glycerin	Alleviate adverse reactions caused by chemotherapy drugs.It should prevent leukopenia caused by chemotherapy bone marrow suppression and improve the patient’s, And improve the quality of life Mo, (2010); Luo et al., (2019)	23	1,113	702
SFI	Red Ginseng, Epibolus	increase the clinical efficacy through inducing the cancer cell apoptosis, inhibiting cell proliferation metastasis, and upregulating tumor immunity Cao et al., (2017)	14	566	359
SI	Red Ginseng, Ophiopogon japonicus, Schisandra chinensis	improving quality of life in patients, immunomodulating action Lu, (2011)	11	511	220
SMI	Red Ginseng, Ophiopogon japonicus	activating the body’s immune system, improving the hematopoietic function of bone marrow, regulating angiogenesis and inhibiting the growth of tumor cells Cheng et al., (2021); Zhong et al., (2020)	21	1,013	537
SQFZI	Dangshen, Huangqi	inhibiting cancer growth, promoting apoptosis, increasing chemotherapy sensitivity, and improving immune functions Li et al., (2015); Xiong et al., (2018)	39	990	1913

Note: ADI, Aidi injection; CSI, Chansu injection; DCI, disodium cantharidinate and vitamin B6 injection; DLSI, Delisheng injection; FFKSI, Fufang Kushen injection; HCSI, Huachansu injection; HQI, Huangqi injection; KAI, Kangai injection; KLTI, Kanglaite injection; OOMI, oil of Ophiopogon injection; SFI, Shenfu injection; SI, Shengmai injection; SMI, Shenmai injection; SQFZI, Shenqi Fuzheng injection; XAPI, Xiaoaiping injection; XGDTI, Xianggu Duotang injection.

### 3.2 Characteristics and quality of included studies

**FIGURE 2:**
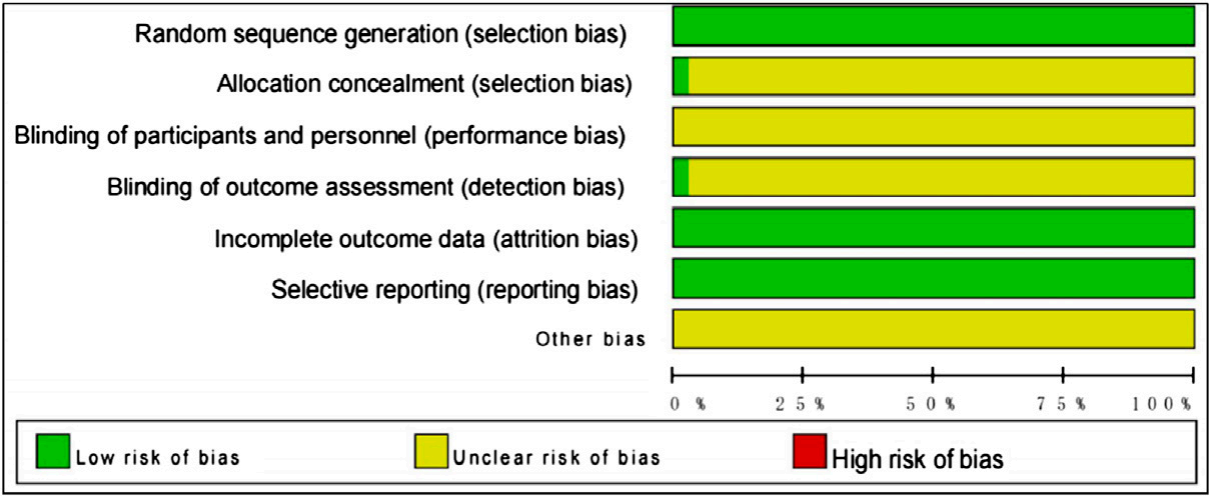
Assessment of risk of bias.

A total of 31,263 patients (15,854 in intervention groups and 15,409 in control groups) were enrolled in the 389 included studies, all of whom were diagnosed with NSCLC in hospital, based on clear diagnostic criteria. The number of people is (males:18,588, females:12,675), and patients had a mean age of 58 years. A total of 3,094 patients received ADI, 499 HCSI, 929 OOMI, 498 DCI, 462 SFI, 788 SMI, 1461 SQFZI, 142 CSI, 284 DLSI, 2373 FFKSI, 244 HQI, 1450 KAI, 1722 KLTI, 370 SI, 702 XGDTI, and 786 XAPI combined with WM therapies. Basic data about the studies analyzed in this paper are listed in (Table 2 and Supplementary Table S2). We assessed the quality of included studies according to the Cochrane Risk of Bias tool. Each evaluation principle was classified as “high risk”, “low risk”, or “unclear”. Of the 389 studies included, 59 RCTs used random number tables for group assignment, 2 used random sampling methods, and 1 applied random assignment by lottery; selection bias associated with random sequence generation in the studies was rated as “low risk”. All included studies reported complete outcome indicators, and their attrition bias was assessed as “low risk”. Detailed results of the risk of bias assessment are shown in Figure 2.

### 3.3 Primary outcomes

#### 3.3.1 DCR

**FIGURE 3:**
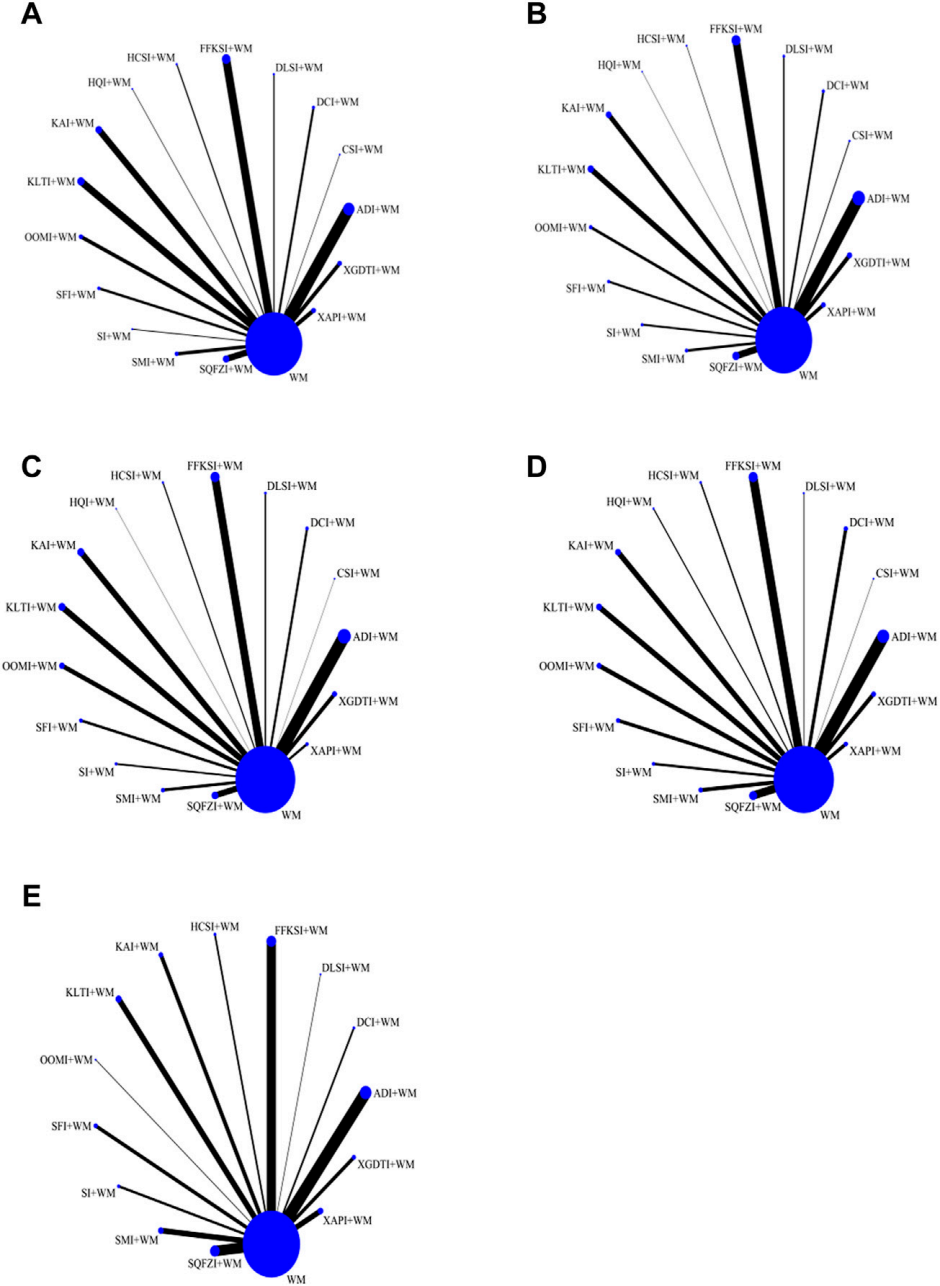
Network graphs for different outcomes. **(A)** Disease control rate. **(B)** Survival quality score. **(C)** Incidence of gastrointestinal adverse reactions. **(D)** Incidence of leukopenia. **(E)** Incidence of thrombocytopenia.

DCR directly reflects the curative effect of treatments on patients and served as the main outcome index in this study. A total of 16 CHIs assessed in 306 RCTs including 25,783 patients, were included in the DCR analysis. Studies of ADI (*n* = 58), CSI (*n* = 3), DCI (*n* = 11), DLSI (*n* = 6), FFKSI (*n* = 42), HCSI (*n* = 6), HQI (*n* = 3), KAI (*n* = 32), KLTI (*n* = 35), OOMI (*n* = 18), SFI (*n* = 12), SI (*n* = 4), SMI (*n* = 16), SQFZI (*n* = 30), XAPI (*n* = 11), and XGDTI (*n* = 19), each combined with WM, were included. A network diagram is shown in Figure 3A. OR values generated by NMA are shown in Supplementary Table S1. DCR values were significantly higher in patients with NSCLC treated with ADI, CSI, DCI, DLSI, FFKSI, HCSI, KAI, KLTI, OOMI, SFI, SMI, SQFZI, XAPI, or XGDTI combined with WM than in those treated with WM alone. There was no significant difference between DCR in patients treated with HQI or SI combined with WM and those receiving WM treatment alone.

**FIGURE 4:**
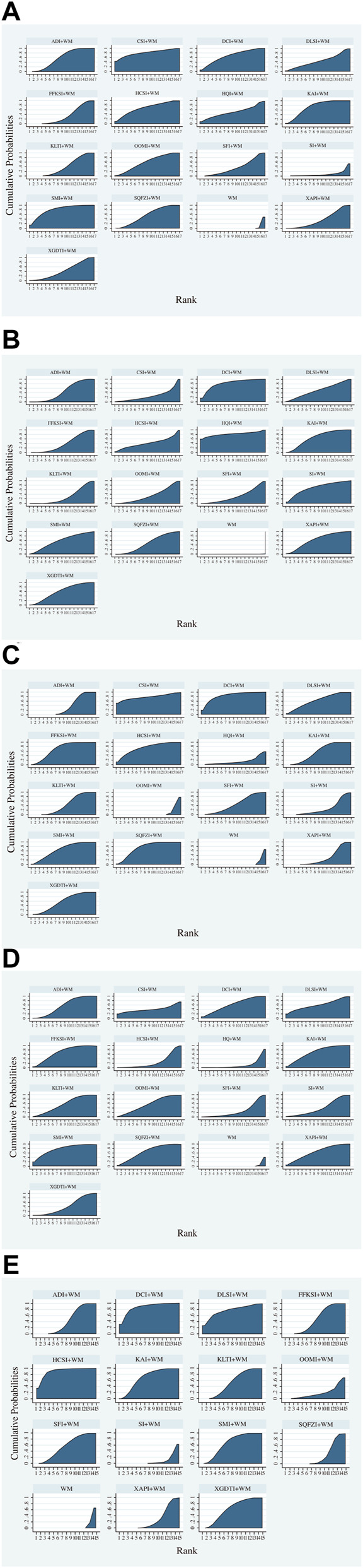
Surface under the cumulative ranking curve area plots for each outcome analyzed (The larger the area under the curve, the higher the ranking and the higher the probability that the CHIs are the best interventions). **(A)** Disease control rate. **(B)** Survival quality score. **(C)** Incidence of gastrointestinal adverse reactions. **(D)** Incidence of leukopenia. **(E)** Incidence of thrombocytopenia.

The results of the SUCRA rankings and probability values (Table 3 and Figure 4A), after ranking the effects of the interventions, indicated that CSI was most likely to improve DCR in patients with NSCLC relative to WM treatment alone (probability, 80.90%).

**TABLE 3:** Surface under the cumulative ranking probabilities analysis (SUCRA) results for five outcome measures.

Interventions	Disease control rate (%)	Survival quality score (%)	Incidence of gastrointestinal adverse reactions (%)	Incidence of leukopenia (%)	Incidence of thrombocytopenia
ADI + WM	58.20	46.70	36.20	58.60	44.60%
CSI + WM	80.90	28.30	79.20	41.90	—
DCI + WM	67.80	82.20	85.50	63.20	87.40%
DLSI + WM	46.30	52.50	64.20	61.50	77.80%
FFKSI + WM	34.80	39.70	71.70	70.90	48.50%
HCSI + WM	69.10	44.20	75.80	26.00	91.30%
HQI + WM	51.50	82.60	16.10	13.10	—
KAI + WM	73.00	67.10	57.40	70.70	70.10%
KLTI + WM	40.20	31.30	44.20	58.40	53.70%
OOMI + WM	60.00	36.10	8.00	58.50	19.70%
SFI + WM	36.90	33.40	46.30	27.70	56.50%
SI + WM	9.40	70.60	27.70	41.50	8.70%
SMI + WM	80.60	60.30	67.70	79.10	67.00%
SQFZI + WM	56.30	49.30	76.20	67.40	26.90%
XAPI + WM	37.90	64.60	29.50	65.50	28.30%
XGDTI + WM	43.80	60.80	58.80	43.10	63.30%
WM	3.30	0.10	5.60	2.90	6.10%

The greater the SUCRA, the greater the likelihood that it will be the best intervention.

#### 3.3.2 Survival quality score

Improvement in quality of survival was assessed for a total of 16 CHIs, 198 RCTs, and 14,700 patients, including studies of ADI (*n* = 40), CSI (*n* = 3), DCI (*n* = 11), DLSI (*n* = 5), FFKSI (*n* = 25), HCSI (*n* = 2), HQI (*n* = 1), KAI (*n* = 16), KLTI (*n* = 18), OOMI (*n* = 9), SFI (*n* = 7), SI (*n* = 6), SMI (*n* = 8), SQFZI (*n* = 21), XAPI (*n* = 12), and XGDTI (*n* = 14), each combined with WM. A network diagram is shown in Figure 3B. OR values generated by NMA are shown in Supplementary Table S2. Compared with the control group treated with WM alone, survival quality scores of patients with NSCLC treated with WM combined with all CHIs were significantly improved.

After ranking the effects of each intervention, the results of the SUCRA ranking and probability values (Table 3 and Figure 4B) indicated that HQI was most likely to improve survival quality score in patients with NSCLC, compared with controls treated with WM alone (probability, 82.60%).

### 3.4 Secondary outcomes

#### 3.4.1 Incidence of GI adverse reactions

A total of 16 CHIs, 222 RCTs, and 18,720 patients were included in analysis of GI adverse reactions, comprising studies of ADI (*n* = 45), CSI (*n* = 1), DCI (*n* = 8), DLSI (*n* = 5), FFKSI (*n* = 30), HCSI (*n* = 4), HQI (*n* = 1), KAI (*n* = 22), KLTI (*n* = 22), OOMI (*n* = 16), SFI (*n* = 9), SI (*n* = 6), SMI (*n* = 10), SQFZI (*n* = 21), XAPI (*n* = 8), and XGDTI (*n* = 14), each combined with WM. A network diagram is shown in Figure 3C. The OR values generated by NMA are presented in Supplementary Table S3. The rate of GI adverse reactions in patients with NSCLC treated with WM combined with ADI, DCI, CSI, DLSI, FFKSI, HCSI, KAI, KLTI, HQI, SFI, SMI, SQFZI, XAPI, or XGDTI was significantly lower than that in patients treated with WM alone. There was no significant difference in GI adverse events between patients receiving OOMI or SI combined with WM and those treated with WM alone.

After ranking the effects of each intervention, the results of the SUCRA rankings and probability values (Table 3 and Figure 4C) indicated that DCI was most likely to reduce the incidence of leukopenia in patients with NSCLC relative to WM treatment alone (probability, 85.50%).

#### 3.4.2 Incidence of leukopenia

Incidence of leukopenia was analyzed for a total of 16 CHIs, in 198 RCTs, and 16,187 patients, comprising studies on ADI (*n* = 37) CSI (*n* = 1), DCI (*n* = 10), DLSI (*n* = 2), FFKSI (*n* = 27), HCSI (*n* = 4), HQI (*n* = 3), KAI (*n* = 16), KLTI (*n* = 16), OOMI (*n* = 13), SFI (*n* = 10), SI (*n* = 7), SMI (*n* = 11), SQFZI (*n* = 23), XAPI (*n* = 6), and XGDTI (*n* = 12), each combined with WM. A network diagram is shown in Figure 3D. The OR values generated by NMA are presented in Supplementary Table S4. The incidence of leukopenia in patients with NSCLC treated with a combination of WM and ADI, DCI, OOMI, DLSI, FFKSI, HCSI, KAI, KLTI, SFI, SI, SMI, SQFZI, XAPI, or XGDTI was significantly lower than that in patients treated with WM alone. HQI or CSI combined with WM did not significantly alter the incidence of leukopenia relative to WM treatment alone.

After ranking the effects of each intervention, the results of the SUCRA rankings and probability values (Table 3 and Figure 4D) indicated that SMI was most likely to reduce leukopenia incidence in patients with NSCLC compared with WM alone, with a probability of 79.10%.

### 3.5 Incidence of thrombocytopenia

Analysis of the incidence of thrombocytopenia included a total of 14 CHIs, 113 RCTs, and 12,648 patients in studies of ADI (*n* = 21), DCI (*n* = 3), DLSI (*n* = 1), FFKSI (*n* = 17), HCSI (*n* = 3), KAI (*n* = 7), KLTI (*n* = 10), OOMI (*n* = 1), SFI (*n* = 6), SI (*n* = 4), SMI (*n* = 9), SQFZI (*n* = 17), XAPI (*n* = 5), and XGDTI (*n* = 6), each combined with WM. A network diagram is shown in Figure 3E. OR values generated by NMA are presented in Supplementary Table S5. The incidence of leukocytopenia in patients with NSCLC treated with a combination of WM and ADI, DCI, HQI, DLSI, CSI, FFKSI, HCSI, KAI, KLTI, SFI, SMI, SQFZI, XAPI, or XGDTI was significantly lower than that in patients treated with WM alone; there was no significant difference between patients treated with OOMI or SI combined with WM and those receiving WM alone.

After ranking the effects of each intervention, the results of the SUCRA rankings and probability values (Table 3 and Figure 4E) indicated that HCSI was most likely to reduce the incidence of thrombocytopenia in patients with NSCLC compared with WM alone (probability, 91.30%).

### 3.6 Cluster analysis

**FIGURE 5:**
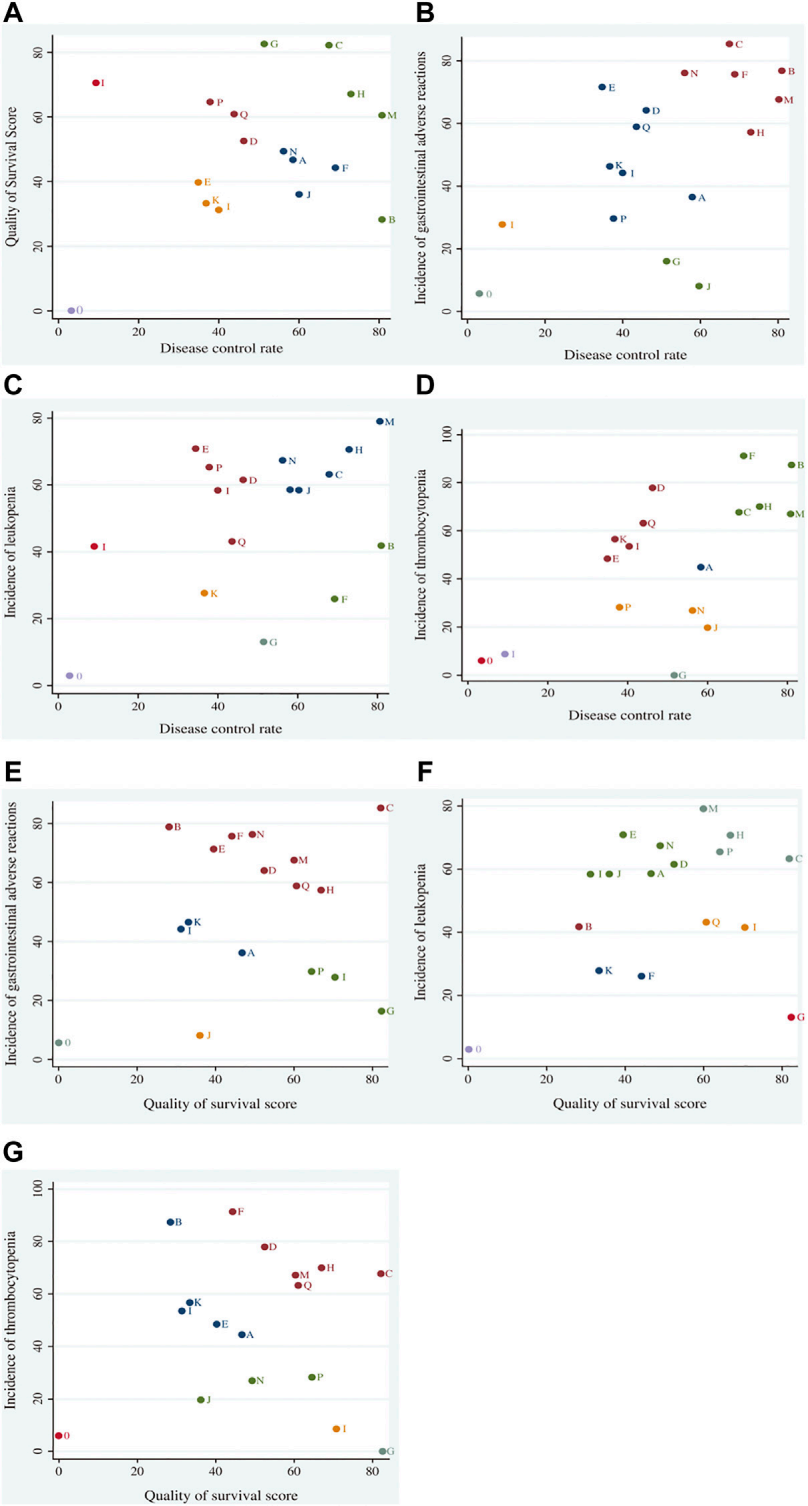
Cluster analysis plots for outcomes. Cluster analysis plot of: **(A)** disease control rate (DCR) and survival quality score, **(B)** DCR and incidence of gastrointestinal adverse reactions, **(C)** DCR and incidence of leukopenia, **(D)** DCR and incidence of thrombocytopenia, **(E)** survival quality score and incidence of gastrointestinal adverse reactions, **(F)** survival quality score and incidence of leukopenia, and **(G)** survival quality score and incidence of thrombocytopenia. Interventions located in the upper right corner indicate optimal therapies for two different outcomes, as follows: A, adi + wm; B, csi + wm; C, dci + wm; D, dlsi + wm; E, flksi + wm; F, hcsi + wm; G, hqi + wm; H, kai + wm; I, klti + wm; J, oomi + wm; K, sfi4-wm; L, si + wtn; M, smi + win; N, sqfzi + wm; 0, wm; P, xapi + wm; Q, xgdti + wm.

Cluster analysis based on SUCRA is illustrated in Figure 5. First, cluster analysis was conducted on DCR and survival quality score. Among eligible treatments, SMI + WM and CSI + WM achieved superior effects over the others in improving DCR and survival quality score, while WM alone ranked toward the bottom. Next, cluster analyses were performed on DCR, survival quality score, and other outcomes. The results revealed that SMI + WM, DCI + WM, and HQI + WM were the highest ranked among the eligible interventions.

### 3.7 Publication bias

**FIGURE 6:**
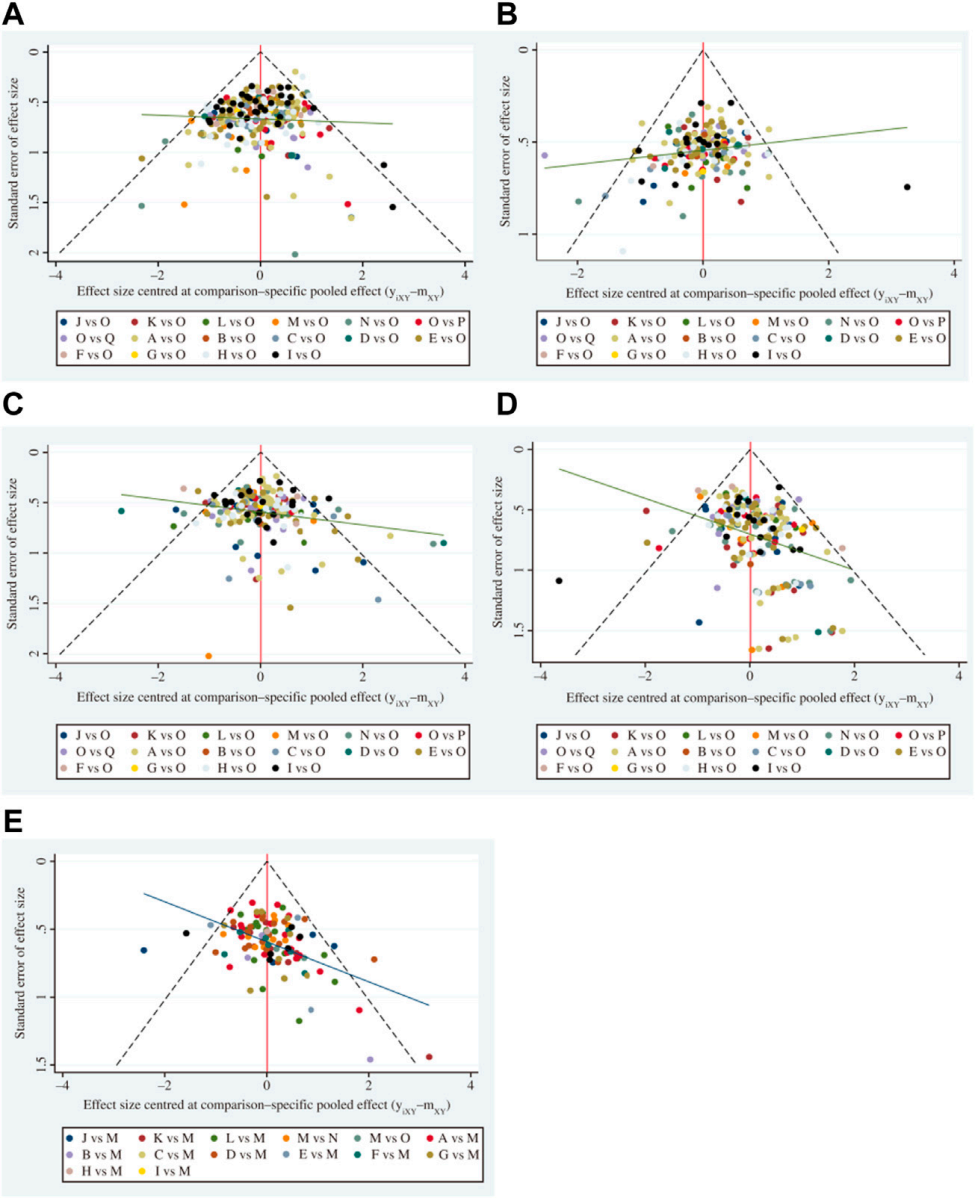
Funnel plots (A comparison-adjusted funnel plot was used to assess potential publication bias. If points on both sides of the midline in the funnel diagram were symmetric, which meant the comection guideline was at right angles to the midline, it was considered indicative of no significant publication bias). **(A)** Disease control rate. **(B)** Survival quality score. **(C)** Incidence of gastrointestinal adverse reactions. **(D)** Incidence of leukopenia. **(E)** Incidence of thrombocytopenia. A, adi + wm; B, csi + wm; C, dci + wm; D, dlsi + wm; E, fflcsi + wm; F, hcsi + wm; G, hqi + wm; H, kai + wm; I, klti + wm; J, oomi + wm; K, sfi + wm; L, si + wm; M, smi + wm; N, sqfzi + wm; 0, win; P, xapi + wm; Q, xgdti + wm.

To assess whether the primary results of this study were affected by reporting bias, we generated Acomparison-adjusted funnel plot. The points on both sides of the center line of the funnel plot were basically symmetrical from left to right; therefore, we assumed that there was no small sample effect. There was an angle between the correction guideline and the centerline, suggesting that our findings may have been influenced by publication bias to some extent (Figure 6).

## 4 Discussion

The severity of NSCLC has been widely recognized due to its high mortality rates and heavy economic burden (Bray et al., 2018; Miller et al., 2022). Currently, a combination of CHIs and WM is widely adopted in China and has achieved the desired efficacy (Cao et al., 2017; Chen, 2018; Duan et al., 2018; Wang J et al., 2018; Cao et al., 2019; Wang et al., 2019; Feng et al., 2020; Zhu et al., 2022). As aim of this study was to supplement the optimal strategy of NSCLC treatment and to strengthen additional insights for clinical practice in the future, this NMA incorporated 389 RCTs, which included 31,263 patients, comparing the efficacy of Sixteen CHIs combined with WM *versus* WM alone. According to the results of the cluster analysis and the SUCRA, all eligible CHIs combined with WM were associated with a more beneficial effect than WM alone. Moreover, SMI + WM and DCI + WM are most likely the optimal CHIs to improve disease control rates, survival quality score, and reduce adverse effects. Hence, the efficacy of SMI + WM and DCI + WM should be considered for patients with NSCLC. However, according to the results of the ORs, there was no significant difference between DCR in patients treated with HQI or SI combined with WM and those receiving WM treatment alone. It is worth noting that there are major differences in the numbers of males and females analyzed in this current NMA. Therefore, clinical treatment decisions should be cautious guided by the specific situation and the clinicians experience. The OR and SUCRA values for some treatment strategies generated in this study were very close; therefore, despite clear advantages over other treatment strategies, they do not represent definitive treatment strategy choices in clinical care.

Shenmai injection (SMI), is derived from a well-known traditional Chinese formula, Shendong Yin, in which the primary pharmacological activity constituents are ginsenosides and Ophiopogon (Liu et al., 2018; Xu et al., 2019; Nag et al., 2012; Chen et al., 2021). Several pharmacological studies reported that SMI has antitumor efficacy and is effective in regulating immune function, enhancing body immunity, and reducing the side effects of chemotherapy (Sun et al., 2020; Li S. Y., 2019; Fang et al., 2018). Ginsenoside Rg3 (the preparation named SMI), a principal pharmacological component of Ginseng, has the potential to reverse drug resistance, inhibit the proliferation of NSCLC cells, and protect DNA integrity (Jiang et al., 2017; Liu L et al., 2019). The undelying mechanism may be that they can attenuate the resistance of cisplatin in lung cancer by inhibiting Akt and NF-KB, resuming immunity, and regulating DNA damage in NSCLC cells by activating the VRK1/P53BP1 pathway (Jiang et al., 2017; Liu T et al., 2019). These findings reveal the impact of ginsenoside Rg3 on DNA damage and downregulating PD-L1, and it opens a new window for developing new drugs based on ginsenoside Rg3 and presents a foundation for developing new therapeutic strategies for cancers. Sodium cantharidinate (SCA), a semi-synthetic derivative of cantharidin, is chemically synthesized from cantharidin and sodium hydroxide (molecular formula, C_10_H_12_Na_2_O_5_). SCA (the preparation named DCI) has the potential to enhance immune function and inhibit the adhesion, invasion, and metastasis of tumor cells (Wang G et al., 2018; Tao et al., 2017; Wen et al., 2013; Wu et al., 2021). Their an mechanisim may be that they can promote the proliferation of T lymphocytes, secrete the cytokine interleukin-2, inhibits the secretion of interleukin-8, downregulates the protein expression of VEGF and MMP-9, restrain the formation of new blood vessels, and control tumor cell adhesion (Tao et al., 2017; Wen et al., 2013; Wu et al., 2021). In addition, SCA could reduce the hematopoietic system toxicity of chemoradiotherapy by shortening the bone marrow maturation, releasing leukocytes’ time, and promoting the differentiation of hematopoietic stem cells into granulocyte/monocyte progenitor cells, as well as increasing white blood cell counts (Wu et al., 2021). Chansu injection (CSI) combined with WM is considered as the best intervention for improving the Disease Control Rate (DCR). Pharmacological studies have revealed that it has good anti-tumor and anti-inflammatory effects, possibly due to its main active ingredient-Toad (Su et al., 2001). The antitumor mechanism of this combination strategy may be that they can induce apoptosis of A549 cells, suppress the survivin mRNA and protein, and increase caspase-3 activity (Morishita et al., 1992; Wang et al., 2009; Wang et al., 2012).

The safety of CHIs should also be evaluated alongside their effectiveness. Although the incidences of ADRs/ADEs were low in this NMA, approximately two-thirds of eligible RCTs did not report ADRs/ADEs, implying that these analyses did not take the safety issue seriously. While describing ADRs/ADEs, this NMA observed that an appropriate course of treatment is essential in treatment. Moreover, appropriate dosage, solution, and syndrome differentiation should also be emphasized.

The present study has some limitations that should be considered when interpreting the findings: 1) The methodological quality of the included studies was not very high. Only 62 of the 389 RCTs described the correct generation of random sequences and no studies mentioned allocation concealment or blinding. 2) The included studies spanned a relatively long period of time and were published in Chinese journals, and their findings may not be fully generalizable to other locations. 3) Most included RCTs compared CHIs combined with WM for treatment of NSCLC, and there was a lack of direct comparisons of two or more CHIs. 4) The sample sizes included in the RCTs varied in size and significant differences may not be detected by studies with small sample sizes. If the sample size is increased, to balance the number of RCTs targeting different types of CHI could improve the statistical power of the data and the credibility of the NMA. 5) Most relevant RCTs did not report the CHIs dosage and ADRs/ADEs, In terms of the above limitations, more rigorous RCTs with high quality are needed to verify the value of CHIs combined with WM for patients with NSCLC.

Although this study has some limitations, it is the first to comprehensively assess the efficacy and safety of CHIs in combination with WM for treatment of NSCLC, using a NMA to rank DCR, survival quality scores, incidence of GI adverse events, incidence of leukopenia, and incidence of thrombocytopenia. This NMA provides clinicians with a detailed comparison of common treatment strategies and may provide a reference for clinical application.

## 5 Conclusion

Overall, this NMA provides a comprehensive and integrated evaluation and summary of the findings using CHIs for treatment of NSCLC. The current evidence indicates that CHIs combined with WM might have a more beneficial effect on NSCLC patients than WM alone, particularly SMI + WM and DCI + WM. It is imperative for clinicians to consider the efficacy of CHIs when diagnosing and treating patients. Future studies should include high quality RCTs, and real-world data are needed to confirm and support the findings of this NMA.

## Data Availability

The original contributions presented in the study are included in the article/Supplementary Material, further inquiries can be directed to the corresponding author.

## Abbreviations

95% CI, 95% confidence interval; ADI, Aidi injection; CHIs, Chinese herbal injections; CM, Chinese medicine; CSI, Chansu injection; DCI, disodium cantharidinate and vitamin B6 injection; DCR, disease control rate; DLSI, Delisheng injection; FFKSI, Fufang Kushen injection; GI, gastrointestinal; HCSI, Huachansu injection; HQI, Huangqi injection; INPLASY, International platform of registered systematic review and meta-analysis protocols; KAI, Kangai injection; KLTI, Kanglaite injection; KPS, Karnofsky performance scale; NSCLC, non-small cell lung cancer; NMA, network meta-analysis; OOMI, oil of Ophiopogon injection; OR, odds ratio; RCT, randomized controlled trial; SFI, Shenfu injection; SI, Shengmai injection; SMI, Shenmai injection; SQFZI, Shenqi Fuzheng injection; SUCRA, surface under the cumulative ranking curve area; WM, western medicine; XAPI, Xiaoaiping injection; XGDTI, Xianggu Duotang injection.
