# Supplementary material for: Comparative efficacy of various CHIs combined with western medicine for non-small cell lung cancer: A bayesian network meta-analysis of randomized controlled trials
Source: Front Pharmacol. 2022 Nov 10;13:1037620. doi: 10.3389/fphar.2022.1037620 (PMC9686447; doi:10.3389/fphar.2022.1037620)
Supplement: Supplementary file 1 [file DataSheet1.PDF]

Section and Topic	Item #	Checklist item	Location where item is reported
**TITLE**			
Title	1	Identify the report as a systematic review.	1
**ABSTRACT**			
Abstract	2	See the PRISMA 2020 for Abstracts checklist.	13-46
**INTRODUCTION**			
Rationale	3	Describe the rationale for the review in the context of existing knowledge.	49-66
Objectives	4	Provide an explicit statement of the objective(s) or question(s) the review addresses.	67-94
**METHODS**			
Eligibility criteria	5	Specify the inclusion and exclusion criteria for the review and how studies were grouped for the syntheses.	97-105
Information sources	6	Specify all databases, registers, websites, organisations, reference lists and other sources searched or consulted to identify studies. Specify the date when each source was last searched or consulted.	107-110
Search strategy	7	Present the full search strategies for all databases, registers and websites, including any filters and limits used.	110-116
Selection process	8	Specify the methods used to decide whether a study met the inclusion criteria of the review, including how many reviewers screened each record and each report retrieved, whether they worked independently, and if applicable, details of automation tools used in the process.	119-128
Data collection process	9	Specify the methods used to collect data from reports, including how many reviewers collected data from each report, whether they worked independently, any processes for obtaining or confirming data from study investigators, and if applicable, details of automation tools used in the process.	143-158
Data items	10a	List and define all outcomes for which data were sought. Specify whether all results that were compatible with each outcome domain in each study were sought (e.g. for all measures, time points, analyses), and if not, the methods used to decide which results to collect.	130-138
	10b	List and define all other variables for which data were sought (e.g. participant and intervention characteristics, funding sources). Describe any assumptions made about any missing or unclear information.	122-128
Study risk of bias assessment	11	Specify the methods used to assess risk of bias in the included studies, including details of the tool(s) used, how many reviewers assessed each study and whether they worked independently, and if applicable, details of automation tools used in the process.	151-158
Effect measures	12	Specify for each outcome the effect measure(s) (e.g. risk ratio, mean difference) used in the synthesis or presentation of results.	167-169
Synthesis methods	13a	Describe the processes used to decide which studies were eligible for each synthesis (e.g. tabulating the study intervention characteristics and comparing against the planned groups for each synthesis (item #5)).	160-181
	13b	Describe any methods required to prepare the data for presentation or synthesis, such as handling of missing summary statistics, or data conversions.	160-181
	13c	Describe any methods used to tabulate or visually display results of individual studies and syntheses.	160-181
	13d	Describe any methods used to synthesize results and provide a rationale for the choice(s). If meta-analysis was performed, describe the model(s), method(s) to identify the presence and extent of statistical heterogeneity, and software package(s) used.	160-181
	13e	Describe any methods used to explore possible causes of heterogeneity among study results (e.g. subgroup analysis, meta-regression).	160-181
	13f	Describe any sensitivity analyses conducted to assess robustness of the synthesized results.	160-181
Reporting bias assessment	14	Describe any methods used to assess risk of bias due to missing results in a synthesis (arising from reporting biases).	160-181
Certainty assessment	15	Describe any methods used to assess certainty (or confidence) in the body of evidence for an outcome.	160-181

Section and Topic	Item #	Checklist item	Location where item is reported
**RESULTS**			
Study selection	16a	Describe the results of the search and selection process, from the number of records identified in the search to the number of studies included in the review, ideally using a flow diagram.	185-196
	16b	Cite studies that might appear to meet the inclusion criteria, but which were excluded, and explain why they were excluded.	185-196
Study characteristics	17	Cite each included study and present its characteristics.	198-204
Risk of bias in studies	18	Present assessments of risk of bias for each included study.	204-210
Results of individual studies	19	For all outcomes, present, for each study: (a) summary statistics for each group (where appropriate) and (b) an effect estimate and its precision (e.g. confidence/credible interval), ideally using structured tables or plots.	204,210
Results of syntheses	20a	For each synthesis, briefly summarise the characteristics and risk of bias among contributing studies.	213-277
	20b	Present results of all statistical syntheses conducted. If meta-analysis was done, present for each the summary estimate and its precision (e.g. confidence/credible interval) and measures of statistical heterogeneity. If comparing groups, describe the direction of the effect.	213-277
	20c	Present results of all investigations of possible causes of heterogeneity among study results.	213-277
	20d	Present results of all sensitivity analyses conducted to assess the robustness of the synthesized results.	213-277
Reporting biases	21	Present assessments of risk of bias due to missing results (arising from reporting biases) for each synthesis assessed.	279-290
Certainty of evidence	22	Present assessments of certainty (or confidence) in the body of evidence for each outcome assessed.	279-290
**DISCUSSION**			
Discussion	23a	Provide a general interpretation of the results in the context of other evidence.	292-312
	23b	Discuss any limitations of the evidence included in the review.	313-312
	23c	Discuss any limitations of the review processes used.	313-327
	23d	Discuss implications of the results for practice, policy, and future research.	329-333
**OTHER INFORMATION**			
Registration and protocol	24a	Provide registration information for the review, including register name and registration number, or state that the review was not registered.	44-46
	24b	Indicate where the review protocol can be accessed, or state that a protocol was not prepared.	
	24c	Describe and explain any amendments to information provided at registration or in the protocol.	
Support	25	Describe sources of financial or non-financial support for the review, and the role of the funders or sponsors in the review.	343-344
Competing interests	26	Declare any competing interests of review authors.	335-336
Availability of data, code and other materials	27	Report which of the following are publicly available and where they can be found: template data collection forms; data extracted from included studies; data used for all analyses; analytic code; any other materials used in the review.	

From: Page MJ, McKenzie JE, Bossuyt PM, Boutron I, Hoffmann TC, Mulrow CD, et al. The PRISMA 2020 statement: an updated guideline for reporting systematic reviews. BMJ 2021;372:n71. doi: 10.1136/bmj.n71

For more information, visit: http://www.prisma-statement.org/

TABLE 1 | More details about the product information of 16 CHIs

Chinese herbal injection(Name of the formulation)	Species / Raw materials	Characteristic	Therapeutic claims in TCM	Indications	Adverse drug reactions
Addi Injection	Zanthoxylum, ginseng, astragalus	Light brown clear liquid	Clearing heat and removing toxins, eliminating blood stasis and dispersing nodules.	Used for liver cancer, lung cancer, rectal cancer, malignant lymphoma, gynecological malignancies, etc.	Occasionally, patients may experience reactions such as flushing, urticaria and fever when applying this product for the first time, and very few patients may experience palpitations, chest tightness, nausea, etc.
Huachansu Injection	Dried toad skin extract	Detoxification, anti-swelling, pain relief	Detoxification, anti-swelling, pain relief.	For intermediate and advanced tumors, chronic hepatitis B and other conditions	Urticaria, dermatitis, etc.
Oil of Ophiopogon Injection	Ophiopogon oil, soy phospholipids	Milky white homogeneous milky liquid	NA	Adjuvant for lung cancer, lung cancer brain metastasis, gastrointestinal tumors and liver cancer	Occasional reactions of greasiness, nausea, anorexia and other digestive discomfort
Disodium Cantharidinate and Vitamin B6 Injection	Sodium zebrachoate, vitamin	Colorless clear liquid	NA	For the treatment of advanced primary liver cancer, and advanced lung cancer	Local phlebitis
Shenfu Injection	Red ginseng, epimedium, excipients are polysorbate	Light yellow or light yellow-brown clear liquid	Returning yang to rescue rebellion, benefiting qi and fixing detachment.	It is mainly used for syncope (infectious, blood loss, fluid loss shock, etc.) caused by Yang deficiency (Qi deficiency); it can also be used for palpitation, palpitation, coughing, gastric pain, diarrhea, paralysis, etc.	Tachycardia, allergic reactions, skin rash, dizziness and headache, erratic reflexes, tremor, dyspnea, nausea, visual abnormalities, abnormal liver function, urinary retention, etc.
Shenmai Injection	Red ginseng, maitake, and the excipients are polysorbate, sodium bisulfite, sodium chloride	Light yellow to light brown clear liquid	Benefiting Qi and fixing deficiency, nourishing Yin and generating body fluid, generating pulse.	It is used for the treatment of shock, coronary heart disease, viral myocarditis, chronic pulmonary heart disease and granulocytopenia in the type of deficiency of both qi and yin. It can improve the immune function of tumor patients, and has a certain synergistic effect when combined with chemotherapy drugs, and can reduce the toxic side effects caused by chemotherapy drugs.	Heartburn, shortness of breath, tightness in the chest, flushing of the face, etc.

Shenqifuzheng Injection	Radix Codonopsis pilosulae, Astragalus membranaceus, sodium chloride	Yellow liquid	Benefiting Qi and helping with correction	For the adjuvant treatment of advanced lung cancer that is not suitable for radiotherapy or chemotherapy and manifests Qi deficiency; can be used in conjunction with chemotherapy for the adjuvant treatment of lung cancer and gastric cancer that manifests Qi deficiency	Hypothermia, stomatitis, drowsiness
Chansu Injection	Toadstool, the excipients are anhydrous sodium sulfite, sodium chloride, benzyl alcohol	Pale yellow to yellow clear liquid	Clearing heat and detoxifying the body	Anti-tumor adjuvant drugs	Rash-like reaction
Delisheng Injection	Red ginseng, Astragalus membranaceus, raw toadstool, raw zebrano	Light yellow-brown clear liquid	Benefiting Qi, promoting correctness, eliminating symptoms and dispersing knots	It is used for the evidence of Qi deficiency and stagnation in middle and late stage primary liver cancer	Urological irritation symptoms of urinary frequency and urgency may occur
Fufangkushen Injection	Bitter ginseng, white tulip, excipients are polysorbate 80, sodium hydroxide, acetic acid.	Yellowish brown to reddish brown clear liquid	Clearing heat and dampness, cooling the blood and removing toxins, dispersing nodules and relieving pain	Used for cancer pain and bleeding	Nausea, vomiting, fever, chills, abdominal distention and stomach upset
Huangqi Injection	Astragalus	Yellow or brownish-yellow clear liquid	Benefiting Qi and nourishing the vital energy, supporting the righteous and dispelling the evil, nourishing the heart and opening the veins, strengthening the spleen and promoting dampness	It is also used for the prevention and treatment of viral myocarditis, cardiac insufficiency, viral hepatitis, immune deficiency, chronic nephritis and renal failure, bronchial asthma, chronic bronchitis, tumors, etc. It can also be used to support the weak and sickly.	Drug fever, drug rash, injection site redness and swelling
Kangai Injection	Astragalus, ginseng, bitter ginseng	Light yellow to yellowish brown clear liquid	Benefit Qi and support the body's immune function	Primary liver cancer, lung cancer, rectal cancer, malignant lymphoma, gynecologic malignancies; treatment of chronic hepatitis B due to various causes of hypo- and hypo- leukocytosis.	Occasional adverse reactions such as rash, itching, chills, fever, nausea, vomiting, chest tightness, palpitations, etc.
Kanglete Injection	Coix lacryma oil for injection	Light yellow or light yellow-brown clear liquid.	Primary non-small cell lung cancer and primary hepatocellular carcinoma with deficiency of qi and yin, spleen deficiency and dampness that are not suitable for surgery	Biphasic broad-spectrum anti-cancer drug, which can both efficiently inhibit and kill cancer cells and significantly improve the organism's finishing immune function	Chills, fever, mild nausea and reversible elevation of liver transaminases

Shengmai Injection	Red ginseng, maidenhair, schisandra	Light yellow or light yellow-brown clear liquid.	Benefiting Qi and nourishing Yin, restoring the pulse and fixing detachment	Regulates central nervous function, affects metabolism, regulates immune function, stimulates bone marrow proliferation, increases secretion of hematopoietic factors, etc., thus improving the adaptability of the body and reducing the damaging effects of radiotherapy on the hematopoietic system	Flushing, rash, itching, dyspnea, palpitations, cyanosis, drop in blood pressure, laryngeal edema, anaphylaxis, etc.
Xiangguduotang Injection	Shiitake mushroom polysaccharide	Light yellow to yellow emulsion liquid	Benefiting Qi, strengthening the spleen, tonifying the deficiencies and supporting the righteousness	Adjuvant drug for radiotherapy and chemotherapy for chronic type B migratory hepatitis and tumors.	Occasionally, nausea, vomiting, loss of appetite, and
Xiaoaiping Injection	Passing Vine	Brownish yellow clear liquid	Clearing heat and detoxifying the toxin, resolving phlegm and softening hardness	Esophageal cancer, gastric cancer, lung cancer, liver cancer, and can be combined with adjuvant treatment of radiotherapy and chemotherapy.	Skin flushing, rash, itching, dyspnea, palpitations, cyanosis, decreased blood pressure, laryngeal edema, anaphylaxis, etc.

TABLE 2 | Characteristics of the studies included in this meta-analysis.

Study ID	Gender (M/F)	Age (years)	Intervention A (experimental)		Intervention B (control)		Course of treatment (days)	Outcomes
			Number of cases（n）	Treatment measures	Number of cases（n）	Treatment measures		
Cao Jianzhong（2020）	51/30	51.34±7.36	40	GAP+Addi Injection	41	GAP	56	①↑②↑③↓
Cai Songwang（2011）	213/369	38-73	322	DP/GP/NP+Addi Injection	260	DP/GP/NP	84	①↑②↑③↓
Jiang Shun（2012）	38/8	32	23	DP+Addi Injection	23	DP	42	①↑③↓④↓⑤↓
Shi Zixia（2016）	35/27	55.7±6.50	31	TP+Addi Injection	31	TP	84	①↑②↑④↓⑤↓
He Honggui（2017）	39/23	67.84±5.28	31	DP+Addi Injection	31	DP	42	①↑③↓④↓⑤↓
Huang Wenjin（2017）	46/33	58.74±7.68	39	GP+Addi Injection	40	GP	63	①↑④↓⑤↓
Zhu Junlin（2017）	95/73	52.7±7.60	84	DP+Addi Injection	84	DP	42	①↑③↓④↓⑤↓
Ma Yame（2016）	39/29	66.9±10.60	33	GP+Addi Injection	35	GP	42	①↑③↓④↓⑤↓
Hou Jinlan（2017）	43/65	74.47±8.57	54	Gefitinib+Addi Injection	54	Gefitinib	28	①↑
Lin Qi（2008）	42/19	NA	30	DP+Addi Injection	30	DP	28	①↑②↑③↓④↓
Wu Huifang（2012）	44/14	63.2±5.12	30	TP+Addi Injection	28	TP	42	①↑②↑③↓④↓⑤↓
Hu Zhixiong（2005）	65/22	38-76	45	NP+Addi Injection	42	NP	56	①↑②↑
Sun Lihong（2005）	69/30	NA	49	NP+Addi Injection	49	NP	84	①↑②↑③↓④↓⑤↓
Zhang Aiqin（2005）		NA	36	NP+Addi Injection	34	NP	21	①↑②↑
Zhang Hong（2009）	53/29	35-76	41	TP+Addi Injection	41	TP	42	①↑②↑
Luo Xiangjiang（2005）	59/13	58	36	NP+Addi Injection	36	NP	NA	①↑②↑③↓④↓⑤↓
Heng Yuan（2019）	53/11	56.51±4.28	32	GP+Addi Injection	32	GP	NA	①↑③↓④↓
Bian Meiguang（2007）	44/20	30-75	34	TP+Addi Injection	30	TP	56	①↑②↑③↓④↓
Go cheng Min（2013）	52/28	55-77	41	DP+Addi Injection	39	DP	28	①↑③↓④↓

Lin Song Sen（2011）	52/30	32-79	42	DP+Addi Injection	40	DP	42	①↑②↑③↓④↓⑤↓
Cui Honghai（2010）	39/21	38-76	30	DP+Addi Injection	30	DP	42	①↑②↑
Ma Yanhua（2020）	72/62	44-73	67	GC+Addi Injection	65	GC	84	①↑③↓④↓⑤↓
Zou Yinshui（2006）	56/25	35-73	42	GP+Addi Injection	39	GP	96	①↑②↑③↓
Zhao Jing（2019）	55/31	64.02±2.34	43	GP+Addi Injection	43	GP	42	①↑
Xu Yu（2020）	53/37	42-82	45	GP+Addi Injection	45	GP	63	①↑
Feng Xueren（2008）	88/42	38-74	68	GP+Addi Injection	62	GP	42	①↑②↑③↓④↓⑤↓
wen kang（2009）	52/24	32-77	38	GP+Addi Injection	38	GP	42	①↑③↓④↓⑤↓
Wang Yantao（2012）	46/26	32-74	36	GP+Addi Injection	36	GP	42	①↑②↑③↓④↓⑤↓
Hong Yonggui（2010）	82/78	38-70	90	GP+Addi Injection	70	GP	42	①↑②↑③↓④↓⑤↓
Liu Zan（2014）	30/18	35-80	24	GP+Addi Injection	24	GP	42	②↑
Sun dawei（2019）	54/28	42-78	41	GP+Addi Injection	41	GP	126	①↑②↑③↑
Cheng Bin（2014）	78/23	27-74	49	GP+Addi Injection	52	GP	42	①↑②↑③↓⑤↓
Gusheng（2013）	36/32	61-78	34	GP+Addi Injection	34	GP	42	①↑②↑③↓
Sun Jinbo（2012）	42/26	60-83	34	GP+Addi Injection	34	GP	20	①↑②↑③↓④↓
Zhang Shuliang（2012）	124/180	34-72	168	NP+Addi Injection	136	NP	84	①↑③↑④↓⑤↓
Cui Yanguang（2006）	51/35	34-78	47	NP+Addi Injection	39	NP	42	①↑②↑③↓④↓⑤↓
Wang Wanru（2008）	39/21	38-76	30	NP+Addi Injection	30	NP	42	①↑②↑
Liu Qiaozhen（2020）	43/39	43-70	41	TP+Addi Injection	41	TP	21	①↑③↓④↓
Chen Xueming（2007）	39/25	47-72	32	TP+Addi Injection	32	TP	21	①↑②↑④↓
Lin Qi（2008）	41/19	35-72	30	TP+Addi Injection	30	TP	42	①↑②↑③↓④↓
Zhang Jianhui（2006）	32/24	32-76	28	NP+Addi Injection	28	NP	112	①↑②↑③↓④↓⑤↓

Xiao Ya bine（2006）	48/20	65-79	39	GC+Addi Injection	29	GC	42	①↑②↑③↓④↓⑤↓
Wu Fengjian（2007）	65/33	55.5±4.20	49	TP+Addi Injection	49	TP	63	①↑②↑③↓④↓⑤↓
Zhang Lijuan（2018）	36/26	40.6±8.5	31	Gefitinib+Addi Injection	31	Gefitinib	60	①↑③↓
Ou Liping（2012）	27/12	31-76	23	TP+Addi Injection	16	TP	42	①↑②↑③↓④↓⑤↓
Zhao Fuli（2013）	128/52	52-77	90	DP+Addi Injection	90	DP	84	①↑②↑③↓④↓⑤↓
Du Zhixiang（2011）	94/26	46-68	60	DP+Addi Injection	60	DP	56	①↑②↑③↓
Shi Xiuzhong（2010）	47/9	49-72	28	GP+Addi Injection	28	GP	42	①↑②↑③↓
Li Zongxian（2010）	39/33	29-75	36	GP+Addi Injection	36	GP	42	①↑②↑③↓
Wang Yongshun（2008）	33/10	43-78	23	NP+Addi Injection	20	NP	42	①↑②↑
Wang Huaizhang（2004）	76/37	27-72	56	NP+Addi Injection	57	NP	84	①↑④↓
Wu Xianglan（2005）	42/18	59-76	32	NP+Addi Injection	28	NP	42	①↑②↑③↓④↓
Ma Jing（2020）	49/37	40-70	46	MVP+Addi Injection	40	MVP	42	①↑③↓④↓⑤↓
Geng Kaijian（2020）	61/29	45-76	45	GP+Addi Injection	45	GP	112	①↑③↓⑤↓
Wang Chongyang（2011）	51/13	47-79	32	NP+Addi Injection	32	NP	42	①↑③↓④↓
Lu Zhijun（2011）	39/29	41-76	34	GP+Addi Injection	34	GP	42	①↑③↓④↓
Gao Yumei（2008）	42/30	32-76	36	NP+Addi Injection	36	NP	63	①↑③↓④↓
Yang Guang（2008）	56/26	28-77	40	TP+Addi Injection	42	TP	42	①↑②↑③↓④↓⑤↓
Lin Qiu Ju（2007）	54/26	26-77	40	NP+Addi Injection	40	NP	42	①↑②↑③↓④↓
Guo Chunhui（2020）	51/31	60-84	41	PO+Addi Injection	41	PO	84	①↑③↓④↓⑤↓
Shen Qiuju（2019）	45/39	45-80	42	Gefitinib+Addi Injection	42	Gefitinib	63	①↑②↑③↓④↓⑤↓
Wang Yantao（2012）	46/26	32-74	36	GP+Addi Injection	36	GP	42	①↑②↑③↓④↓⑤↓

Su Lili（2016）	41/19	32-75	30	TP+Addi Injection	30	TP	56	①↑③↓④↓
Li Weiwen（2018）	55/31	43-76	43	PN+Addi Injection	43	PN	42	①↑③↓④↓
Yang Yong（2011）	30/16	41-76	23	TP+Addi Injection	23	TP	28	①↑②↓③↓④↓
Wang Dijin（2004）	67/31	35-72	49	NP+Addi Injection	49	NP	56	①↑②↑
Li Dandan（2015）	29/31	38-52	30	TP+Huachansu Injection	30	TP	42	①↑
Yin Xiangqian（2018）	83/37	45-75	60	EP+Huachansu Injection	60	EP	112	①↑
Cao Chen Yu（2009）	28/22	40-75	25	NP+Huachansu Injection	25	NP	63	①↑②↑
Hu Zhanghua（2012）	43/31	36-78	36	TP+Huachansu Injection	38	TP	42	①↑③↓④↓⑤↓
Jin E（2007）	42/18	52-77	32	NC+Huachansu Injection	28	NC	56	①↑③↓④↓⑤↓
Ma Jinli（2011）	114/103	40-73	109	GP+Huachansu Injection	108	GP	84	①↑③↓④↓⑤↓
Dong Jing（2013）	47/39	46-66	46	AP+Huachansu Injection	40	AP	84	①↑②↑
Yao Jun（2018）	121/79	35-74	100	DP+Huachansu Injection	100	DP	63	①↑③↓④↓
Bian Fang（2018）	33/30	26-56	31	GP+Huachansu Injection	31	GP	63	①↑②↑③↓
Duan Huilong（2018）	37/23	60-75	30	DP+Huachansu Injection	30	DP	84	①↑③↓④↓⑤↓
Chen Xi（2007）	41/19	35-72	30	NP+Ophiopogon Oil Emulsion Injection	30	NP	56	①↑②↑③↓④↓
Du Min（2006）	76/37	27-72	56	NP+Ophiopogon Oil Emulsion Injection	57	NP	84	①↑
Wu Yingju（2008）	48/12	18-78	32	NP+Ophiopogon Oil Emulsion Injection	28	NP	30	②↑④↓
Me Zefeng（2008）	40/25	30-72	35	DP+Ophiopogon Oil Emulsion Injection	30	DP	42	①↑③↓④↓
Dong Xilin（2009）	42/26	60-79	34	NP+Ophiopogon Oil Emulsion Injection	34	NP	84	①↑②↑③↓④↓
Cui Hanzhi（2010）	32/18	58.2	25	DP+Ophiopogon Oil Emulsion Injection	25	DP	42	①↑②↑③↓④↓

Yao Yangwei（2010）	57/21	51-76	40	DP+Ophiopogon Oil Emulsion Injection	38	DP	84	①↑③↓④↓
Fu Xiangjian（2009）	40/21	29-71	31	TP+Ophiopogon Oil Emulsion Injection	30	TP	42	①↑②↑③↓④↓⑤↓
Cao Yang（2013）	40/32	42-71	36	GP+Ophiopogon Oil Emulsion Injection	36	GP	42	①↑②↑③↓④↓⑤↓
Xie Weibo（2013）	70/50	30-72	45	TP+Ophiopogon Oil Emulsion Injection	30	TP	42	①↑②↑③↓④↓
Xie Weibo（2013）		30-72	45	GP+Ophiopogon Oil Emulsion Injection	30	GP	42	①↑②↑③↓④↓
Wen Lingbo（2013）	31/14	35-70	20	DP+Ophiopogon Oil Emulsion Injection	18	DP	42	①↑③↓④↓
Xie Weibo（2014）	83/43	42-88	63	GP+Ophiopogon Oil Emulsion Injection	63	GP	120	①↑②↑③↓④↓
Zhang Yinzi（2014）	61/29	54-80	45	DP+Ophiopogon Oil Emulsion Injection	45	DP	84	①↑③↓
Liu Yuhe（2014）	54/26	29-73	40	TP+Ophiopogon Oil Emulsion Injection	40	TP	42	①↑②↑③↓④↓
Cai Jian（2015）	64/48	42-78	56	DP+Ophiopogon Oil Emulsion Injection	56	DP	56	①↑②↑③↓
Wang Nan（2016）	39/19	60.60-70.40	29	TP+Ophiopogon Oil Emulsion Injection	29	TP	42	①↑③↓④↓
Zhou Jian（2016）	49/27	52-76	38	DP+Ophiopogon Oil Emulsion Injection	38	DP	84	①↑③↓④↓
Zhu Shuangmei（2016）	42/34	60-73	38	Gefitinib+Ophiopogon Oil Emulsion Injection	38	Gefitinib	60	①↑②↑③↓
Tian Li(2017)	63/33	40-79	48	GP+Ophiopogon Oil Emulsion Injection	48	GP	42	①↑②↑
Meng Qingxin(2019)	43/57	25-75	50	Gefitinib+Ophiopogon Oil Emulsion Injection	50	Gefitinib	28	①↑③↓

Kang Tai Rong(2020)	47/42	42-79	44	DP+Ophiopogon Oil Emulsion Injection	44	DP	84	①↑③↓④↓
Liu Surong(2019)	51/47	45-88	49	GP+Ophiopogon Oil Emulsion Injection	49	GP	42	①↑③↓④↓
Li Yuqing(2016)	42/38	20-76	40	GP+Disodium Cantharidinate Injection	40	GP	56	①↑②↑③↓④↓⑤↓
Chen Miao Ai(2017)	37/25	41-70	31	NP+Disodium Cantharidinate Injection	31	NP	10	①↑③↓④↓
Huang Xiying(2016)	46/40	43-80	43	NP+Disodium Cantharidinate Injection	43	NP	42	①↑③↓④↓
Liu Guojian(2015)	45/15	35-76	30	DP+Disodium Cantharidinate Injection	30	DP	42	①↑③↓④↓
Zeng Hongxue(2020)	27/15	40-74	21	DP+Disodium Cantharidinate Injection	21	DP	63	②↑③↓④↓⑤↓
Dong Li(2014)	55/39	26-75	48	GP+Disodium Cantharidinate Injection	46	GP	180	①↑②↑④↓⑤↓
Chen Yilin(2015)	57/49	61-79	53	GP+Disodium Cantharidinate Injection	53	GP	42	①↑③↓④↓
Du Jingyu(2017)	42/18	52-78	30	GP+Disodium Cantharidinate Injection	30	GP	21	①↑
Chen Rui Shen(2005)	42/21	35-76	32	EP+Disodium Cantharidinate Injection	31	EP	42	①↑②↑④↓
Tang Fuliang(2007)	35/25	40-72	30	NP+Disodium Cantharidinate Injection	30	NP	42	①↑②↑③↓④↓
Chang Yongfang(2008)	51/10	60-76	36	GP+Disodium Cantharidinate Injection	25	GP	42	①↑②↑④↓
Wang Shuzhen(2009)	43/22	30-74	33	TP+Disodium Cantharidinate Injection	32	TP	42	①↑②↑
Wei Hongyan(2010)	35/29	34-75	32	TP+Disodium Cantharidinate Injection	32	TP	42	①↑②↑③↓④↓

Yao Jun(2018)	34/44	40-80	39	AP+Disodium Cantharidinate Injection	39	AP	84	①↑③↓④↓⑤↓
Liu Zhanhua(2004)	30/11	42-72	21	NP+Shenfu Injection	20	NP	56	①↑④↓⑤↓
Lu Xinan(2010)	43/17	40-69	30	NP+Shenfu Injection	30	NP	20	①↑③↓④↓
Hou Anji(2008)	34/12	60-75	25	NP+Shenfu Injection	25	NP	42	①↑②↑③↓
Yang Jing(2016)	45/47	31-67	46	TP+Shenfu Injection	46	TP	42	③↓④↓⑤↓
Gu Jun(2019)	37/23	53-68	30	DP+Shenfu Injection	30	DP	42	①↑③↓④↓
Xie Weiguo(2010)	26/24	60-73	25	NP+Shenfu Injection	25	NP	56	②↑③↓④↓⑤↓
Li Jun(2014)	37/23	53-67	30	NP+Shenfu Injection	30	NP	63	①↑②↑
Zhao Lili(2017)	61/39	52-69	50	NP+Shenfu Injection	50	NP	63	①↑②↑
Gao Yingqin(2012)	52/34	39-73	43	GP+Shenfu Injection	43	GP	42	①↑②↑③↓④↓⑤↓
Bo Maoshu(2014)	41/37	36-77	39	DP+Shenfu Injection	39	DP	42	①↑②↑③↓④↓
Gong Hongwei(2008)	41/19	65-79	30	NP+Shenfu Injection	30	NP	42	①↑④↓⑤↓
Li Jianqiang(2008)	35/19	18-75	26	TP+Shenfu Injection	28	TP	84	①↑
Feng Yibing(2011)	44/19	48. 3 ± 6. 8	31	DP+Shenfu Injection	31	DP	84	①↑③↓④↓
Fan Yan(2019)	40/32	37-69	36	GP+Shenfu Injection	36	GP	84	①↑②↑③↓④↓⑤↓
Luo Lihua(2017)	92/70	32-69	81	DP+Shenmai Injection	81	DP	180	①↑③↓④↓⑤↓
Zheng Qinhong(2017)	45/15	32-69	30	DP+Shenmai Injection	30	DP	42	①↑③↓④↓
Chen Qiang Song(2010)	51/9	29-65	30	NP+Shenmai Injection	30	NP	56	①↑②↑
Lu Qunying(2011)	49/11	46-64	30	NP+Shenmai Injection	30	NP	56	④↓⑤↓
Wang Junli(2014)	41/33	53.6±10.5	37	NP+Shenmai Injection	37	NP	56	①↑②↑③↓④↓⑤↓
Sun Xuan(2015)	59/33	42-75	46	GP+Shenmai Injection	46	GP	42	①↑②↑④↓⑤↓
Zhao Lili(2018)	49/33	41-67	41	GP+Shenmai Injection	41	GP	63	①↑③↓④↓⑤↓

Chen Jingsheng(2018)	49/33	44-67	48	DP+Shenmai Injection	48	DP	84	①↑②↑
Fu Qiang(2012)	42/18	37-79	30	GP+Shenmai Injection	30	GP	63	①↑②↓③↑④↑⑤↑
Shen Liping(2014)	23/27	65-81	25	DP+Shenmai Injection	25	DP	42	①↑④↓⑤↓
Yang Zejiang(2005)	52/8	32-72	30	CAP+Shenmai Injection	30	CAP	56	①↑⑤↓
Zhang Mingkui(2009)	34/18	65-81	27	GP+Shenmai Injection	25	GP	30	①↑②↑③↓④↓⑤↓
Guo Zhifeng(2010)	43/19	32-71	31	TP+Shenmai Injection	31	TP	42	①↑②↑③↓④↓⑤↓
Bian Li(2005)	45/15	32-71	30	NP+Shenmai Injection	30	NP	42	①↑②↑③↓
Li Haijin(2012)	35/15	44-75	25	GP+Shenmai Injection	25	GP	42	①↑③↓④↓⑤↓
Shi Zhiyan(2008)	42/18	42-79	25	NP+Shenmai Injection	25	NP	42	①↑③↓④↓⑤↓
Li Ying(2008)	70/37	38-76	54	GP+Shenmai Injection	53	GP	42	①↑②↑③↓④↓⑤↓
Li Songlin(2005)	50/10	38-75	30	NP+Shenmai Injection	30	NP	42	①↑③↓④↓
Li Rong(2016)	63/60	34-81	73	GP+Shenmai Injection	50	GP	42	①↑
Shangguan Min(2012)	35/15	42-77	25	GP+Shenmai Injection	25	GP	42	①↑
Shan Hui Guo(2014)	44/36	42-77	40	DP+Shenmai Injection	40	DP	42	①↑②↑
Jia Yanling(2012)	98/45	60-77	72	NP+Shen Qi Fu Zheng Injection	71	NP	112	①↑②↑③↓④↓⑤↓
Zhang Fulin(2008)	43/17	36-72	30	TP+Shen Qi Fu Zheng Injection	30	TP	42	①↑②↑③↓④↓⑤↓
Li Yong(2007)	65/22	42-81	44	NP+Shen Qi Fu Zheng Injection	43	NP	112	①↑③↓④↓⑤↓
Gong Zhimin(2008)	48/17	34-74	33	NP+Shen Qi Fu Zheng Injection	32	NP	42	①↑②↑③↓④↓⑤↓
Lu Qiguang(2011)	35/27	65-81	33	EP+Shen Qi Fu Zheng Injection	29	EP	42	①↑②↑④↓⑤↓

Luo Shewen(2007)	39/21	33-75	30	TP+Shen Qi Fu Zheng Injection	30	TP	42	①↑②↑③↓④↓
Luo Shizheng(2006)	33/17	35-74	25	TP+Shen Qi Fu Zheng Injection	25	TP	42	①↑②↑
Yu Qingzhong(2007)	43/19	35-74	30	NP+Shen Qi Fu Zheng Injection	32	NP	40	①↑②↑③↓④↓
Cheng Zhengjun(2017)	31/30	40-78	31	TC+Shen Qi Fu Zheng Injection	31	TC	42	①↑②↑④↓
QiaoShu Lin(2012)	36/24	61.2	30	TP+Shen Qi Fu Zheng Injection	30	TP	42	①↑③↓④↓⑤↓
Ding Chunjie(2012)	42/27	38-70	35	GP+Shen Qi Fu Zheng Injection	35	GP	84	①↑②↑④↓⑤↓
Wan Shuzhen(2009)	45/29	65-81	36	EP+Shen Qi Fu Zheng Injection	36	EP	42	①↑②↑
Ma Chenguang(2013)	35/21	67-83	28	DP+Shen Qi Fu Zheng Injection	28	DP	63	①↑②↑③↓④↓⑤↓
Zhonghao(2011)	46/26	60-78	36	GP+Shen Qi Fu Zheng Injection	36	GP	42	①↑②↑③↓④↓
Ding Pengqiang(2016)	78/42	62-80	60	NP+Shen Qi Fu Zheng Injection	60	NP	42	①↑③↓④↓⑤↓
Chen Yifei(2018)	45/35	42-76	40	NP+Shen Qi Fu Zheng Injection	40	NP	42	①↑
Cui Hanzhi(2010)	36/24	60.5	30	TP+Shen Qi Fu Zheng Injection	30	TP	42	①↑②↑③↓④↓⑤↓
Wang Tingxiang(2014)	60/22	43-78	41	NP+Shen Qi Fu Zheng Injection	41	NP	42	①↑②↑③↓④↓⑤↓
Wang Jingke(2020)	58/22	61.3±5.6	40	GP+Shen Qi Fu Zheng Injection	40	GP	42	①↑③↓
Geng Li(2004)	25/15	25-64	25	NP+Shen Qi Fu Zheng Injection	15	NP	42	①↑②↑
Guo Junlan(2019)	49/39	51-78	44	GP+Shen Qi Fu Zheng Injection	44	GP	63	①↑③↓④↓⑤↓
Liu Yangfan(2009)	51/49	56. 5 ± 6. 70	50	DP+Shen Qi Fu Zheng Injection	50	DP	42	②↑

Wang Weimin(2009)	37/15	32-75	24	DP+Shen Qi Fu Zheng Injection	28	DP	42	①↑②↑③↓
Li Haiting(2019)	53/27	48-77	40	GP+Shen Qi Fu Zheng Injection	40	GP	42	①↑
Gong Yuai(2019)	59/25	58.31 ± 7.60	42	TP+Shen Qi Fu Zheng Injection	42	TP	84	①↑③↓④↓⑤↓
Wang Kun(2007)	26/10	38-75	18	NP+Shen Qi Fu Zheng Injection	18	NP	63	①↑②↑④↓⑤↓
Miao Shu Rong(2010)	61/18	38-71	38	NP+Shen Qi Fu Zheng Injection	41	NP	42	①↑②↑③↓④↓⑤↓
Zheng Jiahui(2009)	52/32	43-76	42	NP+Shen Qi Fu Zheng Injection	42	NP	63	①↑③↓④↓⑤↓
Liao Xiaodong(2015)	58/32	42-74	45	NP+Shen Qi Fu Zheng Injection	45	NP	84	①↑②↑③↓④↓⑤↓
Xu Hongjie(2011)	56/24	38-72	40	GP+Shen Qi Fu Zheng Injection	40	GP	42	①↑②↑③↓④↓⑤↓
Wang Haiming(2013)	34/22	32-73	28	EP+Shen Qi Fu Zheng Injection	28	EP	84	①↑②↑④↓⑤↓
Zhao Zhenyu(2014)	80/22	57.3±8.10	52	GP+Shen Qi Fu Zheng Injection	52	GP	42	①↑③↓④↓⑤↓
Ren Junsheng(2015)	49/35	52-73	42	AP+Shen Qi Fu Zheng Injection	42	AP	42	①↑③↓④↓
Dong Qingfen(2012)	56/34	50. 74 ± 8. 42	45	EP+Shen Qi Fu Zheng Injection	45	EP	42	①↑②↑③↓④↓⑤↓
Shan Hui Guo(2014)	44/36	41-76	40	DP+Shen Qi Fu Zheng Injection	40	DP	42	①↑②↑
Chen Yuan Dong(2010)	36/11	50-79	24	TP+Shen Qi Fu Zheng Injection	24	TP	42	①↑②↑④↓
Yun Xiao Yan(2012)	56/29	45-79	48	NP+Shen Qi Fu Zheng Injection	37	NP	28	①↑③↓④↓
Li Haitao(2012)	44/16	49-82	30	TP+Shen Qi Fu Zheng Injection	30	TP	42	①↑②↑④↓
Mai Haiyan(2010)	62/18	57-70	40	TP+Shen Qi Fu Zheng Injection	40	TP	42	②↑

Chen Qing(2009)	98/45	39-74	31	GP+Chansu Injection	31	GP	40	①↑②↑
Yin Lihua(2007)	35/25	36-69	30	EP+Chansu Injection	30	EP	21	①↑②↑③↓④↓
Zhao Jianqing(2006)	59/24	19-74	47	NP+Chansu Injection	36	NP	56	①↑②↑
Wang Xinmin(2010)	36/26	42-73	34	GP+Chansu Injection	28	GP	42	①↑②↑③↓④↓
Zhang Yu(2007)	44/18	42-71	32	TP+DeLisheng Injection	30	TP	42	①↑②↑③↓
Yang Heping(2006)	73/45	33-71	58	NP+DeLisheng Injection	60	NP	20	①↑②↑③↓④↓
Song Aiying(2006)	48/24	40-71	36	NP+DeLisheng Injection	36	NP	42	①↑③↓④↓⑤↓
Feng Wenna(2008)	44/18	30-76	32	TP+DeLisheng Injection	30	TP	42	①↑②↑③↓
Hao Zefeng(2010)	32/32	30-70	32	TP+DeLisheng Injection	32	TP	42	①↑②↑
Wu Zhengwei(2006)	49/11	45-70	30	TP+DeLisheng Injection	30	TP	56	①↑②↑③↓
Zou Minglei(2009)	44/18	30-76	32	TP+DeLisheng Injection	30	TP	42	①↑②↑③↓
Ma Rui(2009)	44/18	32-70	32	TP+DeLisheng Injection	30	TP	42	①↑②↑③↓
Chen Sheng(2016)	70/50	39-74	60	GP+Fufangkushen Injection	60	GP	50	③↓④↓
Feng Yuan(2013)	40/20	52-76	30	Gefitinib+Fufangkushen Injection	30	Gefitinib	90	①↑
Zhang Juanjuan(2015)	48/36	28-78	42	TP+Fufangkushen Injection	42	TP	63	①↑
Li Yan(2019)	49/30	62-85	39	EP+Fufangkushen Injection	40	EP	112	①↑③↓④↓
Sun Zhuping(2008)	50/15	60-82	47	EP+Fufangkushen Injection	18	EP	112	①↑③↓④↓⑤↓
Dong Jing(2012)	43/37	42-66	40	PD+Fufangkushen Injection	40	PD	84	①↑
Feng Guanghui(2016)	92/36	35-64	64	GP+Fufangkushen Injection	64	GP	56	①↑③↓

Liu Yantong(2010)	39/25	33-75	32	TP+Fufangkushen Injection	32	TP	42	①↑②↑③↓④↓⑤↓
Zhang Haifeng(2019)	83/71	46-81	77	TP+Fufangkushen Injection	77	TP	14	①↑③↓④↓⑤↓
Sang Xinwen(2012)	67/39	32-73	54	NP+Fufangkushen Injection	52	NP	28	①↑②↑
Fang Qiuyan(2009)	82/44	60-79	64	GP+Fufangkushen Injection	62	GP	84	①↑③↓④↓⑤↓
Wang Jingna(2020)	55/31	63.26±2.57	43	GP+Fufangkushen Injection	43	GP	42	①↑
Zhou Haiying(2011)	42/38	34-76	40	GP+Fufangkushen Injection	40	GP	63	①↑③↓④↓⑤↓
Liu Guohui(2011)	98/22	36-73	60	GP+Fufangkushen Injection	60	GP	56	①↑②↑③↓④↓⑤↓
Wang Chaoxia(2009)	49/11	38-73	30	GP+Fufangkushen Injection	30	GP	56	①↑②↑③↓④↓⑤↓
Ding Jianpu(2010)	33/17	42-73	25	DP+Fufangkushen Injection	25	DP	56	①↑②↑
Xiao Peng(2012)	68/44	41-72	56	GP+Fufangkushen Injection	56	GP	42	①↑②↑③↓④↓⑤↓
Zhang Peixin(2008)	50/19	29-73	30	NP+Fufangkushen Injection	20	NP	42	①↑②↑③↓④↓⑤↓
Wang Hongchang(2014)	41/21	43-76	32	PE+Fufangkushen Injection	30	PE	84	①↑②↑
Wang Yibing(2015)	56/62	36-74	56	TP+Fufangkushen Injection	52	TP	42	①↑②↑
Song Wenjie(2021)	55/41	35-70	48	TP+Fufangkushen Injection	48	TP	42	①↑②↑③↓④↓
Wang LeYuan(2009)	58/32	27-72	45	NP+Fufangkushen Injection	45	NP	84	①↑②↑③↓④↓⑤↓
Wang Yanjun(2015)	29/31	56-71	30	TP+Fufangkushen Injection	30	TP	84	①↑②↑④↓⑤↓
Yue Li(2012)	50/35	45-73	43	GP+Fufangkushen Injection	42	GP	42	①↑②↑③↓

Tian Xiuling(2013)	48/28	37-72	40	EP+Fufangkushen Injection	36	EP	42	①↑②↑
Yin Feng(2010)	81/48	37-72	68	NP+Fufangkushen Injection	61	NP	45	①↑②↑③↓
FengLihua (2011)	37/20	38-73	29	TP+Fufangkushen Injection	28	TP	42	①↑②↑③↓④↓⑤↓
Xu Miao(2009)	33/10	60-75	23	GP+Fufangkushen Injection	20	GP	42	①↑②↑③↓④↓
Zhang Peng(2012)	39/37	65-88	38	TP+Fufangkushen Injection	38	TP	56	①↑③↓④↓
Li Shiliang(2017)	49/27	35-72	38	TP+Fufangkushen Injection	38	TP	42	①↑②↑③↓④↓
Wang Hong(2009)	80/64	35-75	76	TP+Fufangkushen Injection	68	TP	42	①↑④↓
Chen Weifeng(2015)	72/34	64. 17 ± 10. 71	53	NP+Fufangkushen Injection	52	NP	42	①↑②↑④↓⑤↓
Zhong Yunchao(2016)	55/25	39-79	40	GP+Fufangkushen Injection	40	GP	42	①↑③↓④↓
Jiang Yan(2015)	30/23	65-85	28	GP+Fufangkushen Injection	25	GP	84	①↑②↑③↓④↓⑤↓
Wang Shengye(2012)	92/36	34-75	64	GP+Fufangkushen Injection	64	GP	28	①↑③↓④↓
Wei Wujie(2021)	55/47	45-76	51	Apatinib+Fufangkushen Injection	51	Apatinib	42	①↑②↑④↓
Hou Chenliang(2019)	35/25	30-72	30	Apatinib+Fufangkushen Injection	30	Apatinib	42	①↑④↓
Pang Dongsheng(2011)	44/18	30-72	32	TP+Fufangkushen Injection	30	TP	42	①↑②↑③↓④↓
Wang Zhan(2010)	72/40	46-70	56	TP+Fufangkushen Injection	56	TP	84	①↑②↑③↓④↓⑤↓
Chen Hao(2012)	46/22	40-76	34	TP+Fufangkushen Injection	34	TP	63	①↑②↑③↓④↓⑤↓
Zhang Li(2009)	49/21	35-69	30	NP+Fufangkushen Injection	40	NP	63	①↑②↑③↓④↓⑤↓

Song Xiaowen(2017)	23/24	36-68	27	NP+Fufangkushen Injection	20	NP	42	①↑②↑③↓
Li Jun(2008)	60/40	32-70	60	NP+Fufangkushen Injection	40	NP	42	①↑②↑③↓
Zhou Qixing(2009)	50/15	70-83	33	NP+Fufangkushen Injection	32	NP	56	①↑②↑③↓④↓
Gu Yulin(2015)	53/25	34-72	39	TP+Fufangkushen Injection	39	TP	48	①↑
Liu Yanru(2013)	51/37	36-74	44	GC+Fufangkushen Injection	44	GC	63	①↑③↓
Chang Peijiang(2015)	49/35	34-76	42	GC+Fufangkushen Injection	42	GC	84	①↑②↑③↓④↓⑤↓
Liu Chang(2015)	81/72	61-80	76	GP+Fufangkushen Injection	76	GP	42	①↑③↓⑤↓
Li Baohua(2010)	58/27	60-82	43	GP+Fufangkushen Injection	42	GP	56	①↑③↓④↓
Shi Weiwei(2009)	32/15	35-74	24	GP+Fufangkushen Injection	23	GP	42	①↑②↑
Song Yang(2014)	40/20	43-73	30	GP+Fufangkushen Injection	30	GP	42	①↑②↑③↓④↓⑤↓
Liu Cunwei(2017)	20/20	37-69	20	GP+Fufangkushen Injection	20	GP	42	①↑
JieJaMi(2015)	38/22	38-65	30	GP+Fufangkushen Injection	30	GP	14	①↑
San Huiliang(2015)	53/37	60-82	45	GP+Fufangkushen Injection	45	GP	82	①↑③↓④↓⑤↓
Zhao Kai(2012)	61/25	52-76	43	GP+Fufangkushen Injection	43	GP	42	①↑
Zou Yuhe(2003)	70/50	39-74	30	MVP+HuangQi Injection	30	MVP	84	①↑②↑
Shi Zhiyan(2003)	52/8	43-73	30	EP+HuangQi Injection	30	EP	56	①↑②↑③↓④↓
Zhong Fanghong(2011)	57/23	36-75	40	NP+HuangQi Injection	40	NP	56	①↑④↓

Li Fuchun(2007)	45/15	45-70	28	NP+HuangQi Injection	29	NP	56	①↑②↑③↓④↓
Zhu Baojiang(2010)	45/15	45-70	35	MVP+HuangQi Injection	34	MVP	56	①↑
Liu Xuemei(2003)	45/15	31-72	30	MVP+HuangQi Injection	30	MVP	84	①↑
Wannianqing(2007)	31/10	40-74	21	NP+HuangQi Injection	20	NP	14	①↑④↓
Wang Haicun(2004)	46/14	31-68	30	VP+HuangQi Injection	30	VP	63	①↑
Wang Zhifeng(2017)	54/22	22-73	38	FP+KangAi Injection	38	FP	42	③↓④↓
Shi Lei(2011)	36/22	34-69	29	TP+KangAi Injection	29	TP	42	①↑②↑③↓
Liang Qi Lian(2009)	69/31	52	51	GP+KangAi Injection	49	GP	112	①↑②↑④↓
Zhang Qianyong(2016)	50/32	52	41	TP+KangAi Injection	41	TP	28	①↑③↓④↓
Liu Shuo(2014)	40/32	18-70	36	TP/NP+KangAi Injection	36	TP/NP	42	①↑②↑③↓④↓⑤↓
Huang Ruiwen(2006)	34/16	35-75	25	NP+KangAi Injection	25	NP	42	①↑②↑③↓④↓⑤↓
Jiang Lin(2011)	44/21	50-78	35	NP+KangAi Injection	30	NP	42	①↑②↑③↓④↓
Wu Xiaowei(2018)	217/109	59.0 ± 8.43	165	TP/GP+KangAi Injection	170	TP/GP	42	①↑
Li Yakun(2019)	45/37	37-78	41	ICOTINIB+KangAi Injection	41	ICOTINIB	30	①↑③↓
Cheng Yao(2020)	49/37	58.03±6.27	43	ICOTINIB+KangAi Injection	43	ICOTINIB	30	①↑③↓
Liang Dengfeng(2014)	23/17	70-83	20	ICOTINIB+KangAi Injection	20	ICOTINIB	365	①↑
Zhang Xinglin(2005)	43/19	35-74	30	NP+KangAi Injection	32	NP	56	①↑②↑③↓④↓
Wen Jiyu(2006)	67/11	24-76	40	NP+KangAi Injection	38	NP	84	①↑②↑
Zhang Mingjuan(2009)	76/44	29-75	60	TP+KangAi Injection	60	TP	42	①↑②↑③↓④↓

Li Jinzhang(2009)	32/24	36-70	28	TP+KangAi Injection	28	TP	42	①↑②↑
Jing Hua(2007)	61/36	36-76	51	NP+KangAi Injection	46	NP	63	①↑③↓④↓
Kang Jianrong(2017)	23/17	34-72	20	CEF+KangAi Injection	20	CEF	63	①↑③↓④↓
Fu Hongxing(2018)	60/40	42-71	50	DP+KangAi Injection	50	DP	42	①↑②↑③↓
Cai Peng(2007)	48/12	18-81	32	NP+KangAi Injection	28	NP	30	①↑②↑
Wu Yaqin(2021)	44/28	41-88	37	GP+KangAi Injection	35	GP	56	①↑③↓④↓⑤↓
Gao Zhihao(2021)	33/21	41-76	27	GP+KangAi Injection	27	GP	84	①↑③↓④↓⑤↓
Tu Jianguo(2021)	40/23	70-85	32	TP+KangAi Injection	31	TP	84	①↑②↑③↓④↓⑤↓
Go Changzheng(2011)	47/17	70-79	32	GP+KangAi Injection	32	GP	42	①↑②↑
Luo Jiahua (2017)	28/26	37-72	27	NP+KangAi Injection	27	NP	42	①↑③↓
Li Aimin(2006)	42/23	32-78	34	NP+KangAi Injection	31	NP	42	①↑②↑④↓
Zhang Shicai(2021)	59/31	60-79	45	GP/TP+KangAi Injection	45	GP/TP	42	①↑②↑③↓④↓
Zhao Jingping(2009)	47/28	46-70	38	DP+KangAi Injection	37	DP	42	①↑②↑③↓④↓
Chen Hanrui(2018)	74/72	64.57 ± 8.75	59	GP/DP+KangAi Injection	77	GP/DP	42	①↑
Ma Ming(2017)	53/29	46-75	40	TP+KangAi Injection	42	TP	84	①↑③↓④↓
Li Caiying(2017)	38/22	35-70	30	TP+KangAi Injection	30	TP	42	①↑②↑③↓④↓
Yang Xiaoyu(2010)	37/23	65-70	30	GP+KangAi Injection	30	GP	84	①↑②↑③↓⑤↓
Zhang Li(2012)	38/32	61-71	35	DP+KangAi Injection	35	DP	21	①↑
Wang Liufei(2012)	46/18	30-76	32	TP+KangAi Injection	32	TP	84	①↑②↑③↓④↓
Gao Yongkang(2019)	32/28	36-70	30	DP+KangAi Injection	30	DP	42	①↑③↓
Yu Faming(2018)	51/29	43-73	40	DP+KangAi Injection	40	DP	42	①↑③↓④↓⑤↓
Zhang Yuxin(2010)	17/13	45-70	15	TP+KangAi Injection	15	TP	30	①↑④↓

Zhang Yanian(2019)	38/22	46-77	32	DP+Bevacizumab+KangAi Injection	28	DP+Bevacizumab	42	①↑③↓
Liu Xiaohong(2016)	62/48	46-79	55	NP+KangLaiTe Injection	55	NP	84	①↑
Zhang Shenglin(2012)	43/17	42-88	30	NP/TP/GP+KangLaiTe Injection	30	NP/TP/GP	42	①↑②↑③↓
Zhao Jingping(2009)	47/28	46-70	38	DP+KangLaiTe Injection	37	DP	42	①↑②↑
Fu Peng(2016)	63/37	31-75	53	NP+KangLaiTe Injection	47	NP	112	①↑
Gui Xuemei(2020)	69/51	32-73	60	GP+KangLaiTe Injection	60	GP	56	①↑③↓④↓
Wang Yuan(2017)	32/40	52. 14 ±2. 01	36	GP+KangLaiTe Injection	36	GP	28	①↑②↑
Wang Xiaoqing(2014)	46/12	37-70	28	NP+KangLaiTe Injection	30	NP	42	①↑③↓④↓⑤↓
Cheng Sanzhen(2020)	97/93	38-76	100	NP+KangLaiTe Injection	100	NP	84	①↑③↓④↓
Li Hongyun(2002)	42/39	55-75	41	GP+KangLaiTe Injection	41	GP	84	①↑
Ye Chengyuan(2019)	54/26	55-74	40	GP+KangLaiTe Injection	40	GP	42	①↑③↓
Chen Wei(2016)	47/41	55-78	44	GP+KangLaiTe Injection	44	GP	84	①↑
Zhang Qi(2011)	36/42	52.4+4.00	39	Gefitinib+KangLaiTe Injection	39	Gefitinib	63	①↑②↑③↓
Zheng Lei(2020)	61/59	31-63	62	GP+KangLaiTe Injection	58	GP	60	①↑③↓④↓
Chen Weiwei(2015)	34/26	36-71	30	NP+KangLaiTe Injection	30	NP	21	①↑②↑③↓④↓
Lu Changdong(2008)	81/48	21-71	68	NP/GP+KangLaiTe Injection	61	NP/GP	63	①↑②↑③↓
Wu Ping(2004)	46/18	32-76	34	NP/MVP+KangLaiTe Injection	30	NP/MVP	42	①↑④↓⑤↓
Wang Yuling(2005)	51/29	38-72	42	NP/MVP/GP/TP+KangLaiTe Injection	38	NP/MVP/GP/TP	42	②↑

Li Min(2019)	29/31	39-70	30	NP+KangLaiTe Injection	30	NP	42	①↑④↓⑤↓
Hou En Cun(2008)	41/27	37-71	34	NP+KangLaiTe Injection	34	NP	42	①↑②↑③↓④↓
Qian Wenjun(2013)	71/19	65. 8±8. 1	45	GP+KangLaiTe Injection	45	GP	120	①↑③↓
Jiang Fusheng(2010)	35/27	51-79	32	TP+KangLaiTe Injection	30	TP	56	②↑③↓④↓⑤↓
Wang Yeya(2013)	55/23	45-77	39	GP+KangLaiTe Injection	39	GP	56	①↑②↑③↓④↓⑤↓
Li Xiangdong(2016)	43/35	35-72	39	NP+KangLaiTe Injection	39	NP	56	①↑
Lu Xiaomin(2016)	28/20	44-71	24	DP+KangLaiTe Injection	24	DP	42	①↑③↓④↓⑤↓
Yang Weijing(2018)	47/39	36-80	43	ICOTINIB+KangLaiTe Injection	43	ICOTINIB	63	①↑②↑③↓
Zhu Yongzhong(2018)	68/32	35-72	50	DP+KangLaiTe Injection	50	DP	42	①↑
Xiao Xuejun(2020)	41/34	50-69	38	TP+KangLaiTe Injection	37	TP	42	①↑③↓
Wang Zhenxing(2007)	62/18	38-71	39	NP+KangLaiTe Injection	41	NP	56	①↑③↓④↓⑤↓
Zhang Hua(2010)	48/14	38-71	30	NP+KangLaiTe Injection	32	NP	42	①↑③↓④↓⑤↓
Li Liqun(2010)	29/13	42-70	22	NP+KangLaiTe Injection	20	NP	42	①↑②↑③↓
Ren Jianhong(2010)	83/44	42-70	64	NP+KangLaiTe Injection	63	NP	42	①↑③↓④↓⑤↓
Wu Xiaohong(2001)	32/8	38-76	21	MVP+KangLaiTe Injection	19	MVP	42	①↑②↑④↓
Han Bei(2018)	127/73	50-78	99	GP+KangLaiTe Injection	101	GP	42	①↑②↑③↓④↓⑤↓
Chen Cheng(2018)	31/29	36-63	30	GP+KangLaiTe Injection	30	GP	21	①↑③↓④↓
Zhang Shuhui(2014)	50/60	29-76	55	Gefitinib+KangLaiTe Injection	55	Gefitinib	63	①↑②↑
Yao Jun(2017)	78/59	64.80 ±7.20	70	GP+KangLaiTe Injection	67	GP	42	①↑③↓④↓⑤↓
Yang Shujun(2003)	NA	40-68	28	NP+KangLaiTe Injection	29	NP	42	①↑②↑

Li Li(2012)	49/29	70-77	38	TP+KangLaiTe Injection	40	TP	63	①↑②↑
Zhang Haiying(2014)	113/91	41-75	102	TP+KangLaiTe Injection	102	TP	42	①↑②↑③↓
Huang Jian(2007)	93/27	32-70	62	TP+ShenMai Injection	58	TP	56	②↑④↓
Wang Rong(2006)	34/18	36-72	26	GP+ShenMai Injection	26	GP	42	①↑②↑③↓④↓⑤↓
Liu Jingying(2014)	54/34	38-70	44	GP+ShenMai Injection	44	GP	42	①↑②↑
Zhang Chunmei(2014)	47/13	38-66	30	GP+ShenMai Injection	30	GP	28	①↑②↑③↓④↓
Liang Cuiwei(2007)	25/13	41-72	19	DP+ShenMai Injection	19	DP	42	①↑②↑③↓
Lu Xinan(2011)	48/12	32-70	30	NP+ShenMai Injection	30	NP	28	②↑③↓④↓
Chen Qiang Song(2011)	42/18	46-69	30	NP+ShenMai Injection	30	NP	56	②↑③↓④↓⑤↓
Wang Dongjian(2012)	35/17	36-75	26	TP+ShenMai Injection	26	TP	14	①↑②↑③↓④↓⑤↓
Li Gongxing(2005)	35/23	40-70	30	GEP/MEP+ShenMai Injection	28	GEP/MEP	42	①↑②↑③↓
Bao Yuhua Bao(2011)	45/16	45-68	31	GP/TP/DP+ShenMai Injection	30	GP/TP/DP	56	②↑③↓④↓⑤↓
Chen Yanzhi(2005)	53/29	39-74	42	NP+ShenMai Injection	40	NP	42	①↑④↓⑤↓
Lu Hong(2007)	38/25	32-70	32	NP+Xiangguduotang Injection	32	NP	42	①↑②↑③↓④↓
Li Qin(2021)	65/39	49-79	52	Gefitinib+Xiangguduotang Injection	52	Gefitinib	63	①↑②↑③↓
Jiang Rongfang(2010)	64/34	26-78	49	GP+Xiangguduotang Injection	49	GP	84	①↑②↑③↓④↓⑤↓
Zhao Wenying(2017)	46/34	26-74	40	NP+Xiangguduotang Injection	40	NP	42	①↑②↑③↓
Zhao Lu(2012)	NA	32-74	34	GP+Xiangguduotang Injection	34	GP	84	①↑②↑

Zhang Haimei(2009)	68/45	42-76	57	TP+Xiangguduotang Injection	56	TP	42	①↑②↑③↓④↓
Wang Wenwu(2006)	65/16	41-70	42	NP+Xiangguduotang Injection	39	NP	42	①↑②↑③↓④↓
Shixin(2007)	55/9	35-69	33	NP+Xiangguduotang Injection	31	NP	42	①↑②↑
Wang Jia Ying(2011)	63/19	50.7 ±3.6	40	NP+Xiangguduotang Injection	42	NP	56	①↑②↑③↓④↓
Chen Jianqiang(2008)	64/28	40-68	47	GP+Xiangguduotang Injection	45	GP	42	①↑②↑③↓④↓
Ding Ning(2012)	53/33	NA	44	NP+Xiangguduotang Injection	42	NP	42	①↑②↑③↓④↓⑤↓
Ma Jinhua(2014)	54/50	33-73	52	NP+Xiangguduotang Injection	52	NP	42	①↑③↓④↓
Dong Ying(2017)	34/18	54.8±4.9	26	GP+Xiangguduotang Injection	26	GP	56	①↑
Han Liang(2012)	29/22	56	25	GP+Xiangguduotang Injection	26	GP	42	①↑③↓④↓
Daidong(2014)	42/26	25-78	34	GP+Xiangguduotang Injection	34	GP	42	①↑②↑③↓④↓⑤↓
Li Zhiying(2009)	38/24	40-68	31	GP+Xiangguduotang Injection	31	GP	42	①↑②↑③↓④↓⑤↓
Zhao Xulin(2011)	39/22	39-73	31	GP+Xiangguduotang Injection	30	GP	56	①↑③↓④↓⑤↓
Chai Manchao(2005)	51/14	43-71	33	GAP/CE+Xiangguduotang Injection	32	GAP/CE	42	①↑③↓④↓
Song Cui Ping(2008)	38/25	43-71	36	NP+XiaoAiPing Injection	36	NP	42	①↑②↑③↓
Wang Wenyu(2009)	37/19	39-78	27	TP+XiaoAiPing Injection	29	TP	42	①↑②↑
Zhang Fengyun(2011)	31/17	50-75	24	GP+XiaoAiPing Injection	24	GP	42	①↑②↑④↓⑤↓
Wang Yantao(2012)	38/18	40-70	28	GP+XiaoAiPing Injection	28	GP	42	①↑②↑③↓④↓⑤↓

Zhang Ruixing(2012)	NA	40-75	35	GP/NP+XiaoAiPing Injection	33	GP/NP	42	①↑②↑③↓④↓⑤↓
Xia Guoan(2013)	51/27	39-77	39	TP+XiaoAiPing Injection	39	TP	42	①↑②↑
Fang Huan(2013)	56/30	41-86	43	GP+XiaoAiPing Injection	43	GP	63	①↑③↓④↓⑤↓
Li Yali(2015)	39/26	39-77	33	TC+XiaoAiPing Injection	32	TC	42	①↑②↑③↓④↓⑤↓
Mei Chaorong(2015)	38/25	38-78	30	TP+XiaoAiPing Injection	33	TP	42	①↑②↑③↓④↓⑤↓
Shen Liwei(2015)	39/17	50-75	28	TP+XiaoAiPing Injection	28	TP	42	①↑②↑④↓⑤↓
Li Qinglin(2016)	54/18	27-74	36	GP+XiaoAiPing Injection	36	GP	42	①↑②↑③↓⑤↓
Gu Ning(2016)	54/24	46-76	39	GP+XiaoAiPing Injection	39	GP	21	①↑②↑
Liu Aifang(2016)	43/29	49-76	36	GP+XiaoAiPing Injection	36	GP	21	①↑②↑
Gao Zhihao(2016)	40/28	53-78	34	TC+XiaoAiPing Injection	34	TC	42	①↑③↓⑤↓
Yao Jun(2016)	49/57	45-75	53	TP+XiaoAiPing Injection	53	TP	42	①↑③↓④↓⑤↓
Song Yu(2016)	46/34	65.3±2.8	40	GP/NP+XiaoAiPing Injection	40	GP/NP	21	①↑④↓⑤↓
Hu Xiaolin(2017)	66/40	32-74	53	GP+XiaoAiPing Injection	53	GP	56	①↑④↓⑤↓
Ye Lu(2017)	38/22	42-62	30	TP+XiaoAiPing Injection	30	TP	21	①↑③↓④↓⑤↓
Jiang Ziyu(2017)	27/23	35-74	25	TP+XiaoAiPing Injection	25	TP	42	①↑
Zhang Jing(2018)	41/35	35-74	38	ICOTINIB+XiaoAiPing Injection	38	ICOTINIB	28	①↑
Li Xuepeng(2018)	53/25	37-72	39	GP+XiaoAiPing Injection	39	GP	42	①↑
Rao Shijun(2018)	48/32	34-75	40	GP+XiaoAiPing Injection	40	GP	42	①↑②↑

Note: ①:Disease control rate ; ②: Quality of Survival Score; ③: Gastrointestinal adverse reactions; ④: Incidence of leukopenia ⑤:Incidence of thrombocytopenia

Symbols: In this table, ↑ Indicates CHIs Combined with WM treatment increase or improvement Disease control rate and Survival Quality Score ; ↓ Indicates CHIs Combined with WM treatment reduction Gastrointestinal adverse reactions; Incidence of leukopenia ;Incidence of thrombocytopenia.

Table S1 Results of the network meta-analysis of DCR.

.

	ADI	CSI	DCI	DLSI	FFKSI	HCSI	HQI	KAI	KLTI	OOMI	WM	SFI	SI	SMI	SQFZI	XAPI	XGDTI
ADI	ADI	1.35 (0.63, 2.95)	1.08 (0.7, 1.67)	0.91 (0.51, 1.64)	0.87 (0.69, 1.11)	1.1 (0.68, 1.78)	0.94 (0.44, 2.02)	1.11 (0.85, 1.45)	0.87 (0.68, 1.12)	1.02 (0.72, 1.45)	0.48 (0.41, 0.57)	0.84 (0.56, 1.25)	0.49 (0.23, 1.07)	1.24 (0.85, 1.81)	1.02 (0.76, 1.36)	0.89 (0.64, 1.22)	0.9 (0.64, 1.29)
CSI	0.74 (0.34, 1.58)	CSI	0.8 (0.34, 1.86)	0.67 (0.26, 1.68)	0.65 (0.3, 1.38)	0.81 (0.34, 1.94)	0.7 (0.24, 1.99)	0.83 (0.37, 1.78)	0.65 (0.29, 1.39)	0.76 (0.33, 1.69)	0.36 (0.17, 0.74)	0.62 (0.27, 1.42)	0.37 (0.13, 1.06)	0.92 (0.4, 2.06)	0.75 (0.34, 1.65)	0.66 (0.29, 1.44)	0.67 (0.3, 1.5)
DCI	0.93 (0.6, 1.43)	1.25 (0.54, 2.97)	DCI	0.84 (0.43, 1.68)	0.81 (0.52, 1.25)	1.02 (0.56, 1.86)	0.87 (0.38, 2.03)	1.03 (0.65, 1.63)	0.81 (0.52, 1.26)	0.95 (0.57, 1.57)	0.45 (0.3, 0.66)	0.78 (0.45, 1.34)	0.46 (0.2, 1.08)	1.15 (0.68, 1.94)	0.94 (0.59, 1.51)	0.82 (0.5, 1.33)	0.84 (0.51, 1.39)
DLSI	1.1 (0.61, 1.97)	1.48 (0.59, 3.81)	1.19 (0.6, 2.35)	DLSI	0.96 (0.53, 1.73)	1.21 (0.59, 2.49)	1.04 (0.41, 2.63)	1.23 (0.67, 2.24)	0.96 (0.53, 1.74)	1.12 (0.59, 2.13)	0.53 (0.3, 0.92)	0.92 (0.47, 1.81)	0.54 (0.21, 1.41)	1.36 (0.71, 2.61)	1.12 (0.61, 2.07)	0.97 (0.52, 1.82)	1 (0.52, 1.89)
FFKSI	1.14 (0.9, 1.46)	1.54 (0.72, 3.38)	1.23 (0.8, 1.92)	1.04 (0.58, 1.88)	FFKSI	1.26 (0.78, 2.06)	1.08 (0.51, 2.32)	1.27 (0.96, 1.69)	1 (0.77, 1.3)	1.17 (0.82, 1.68)	0.55 (0.46, 0.66)	0.96 (0.64, 1.45)	0.56 (0.26, 1.23)	1.41 (0.97, 2.08)	1.16 (0.86, 1.58)	1.01 (0.73, 1.41)	1.04 (0.72, 1.48)
HCSI	0.91 (0.56, 1.47)	1.23 (0.51, 2.98)	0.98 (0.54, 1.79)	0.83 (0.4, 1.71)	0.8 (0.49, 1.29)	HCSI	0.86 (0.36, 2.05)	1.01 (0.61, 1.66)	0.8 (0.48, 1.29)	0.93 (0.54, 1.6)	0.44 (0.28, 0.68)	0.76 (0.43, 1.36)	0.45 (0.19, 1.08)	1.13 (0.64, 1.97)	0.93 (0.55, 1.54)	0.81 (0.47, 1.37)	0.82 (0.47, 1.42)
HQI	1.06 (0.5, 2.25)	1.43 (0.5, 4.15)	1.14 (0.49, 2.65)	0.97 (0.38, 2.43)	0.93 (0.43, 1.98)	1.17 (0.49, 2.78)	HQI	1.18 (0.55, 2.53)	0.93 (0.43, 1.98)	1.08 (0.49, 2.41)	0.51 (0.24, 1.06)	0.89 (0.39, 2.03)	0.52 (0.18, 1.51)	1.31 (0.58, 2.95)	1.08 (0.5, 2.34)	0.94 (0.43, 2.06)	0.96 (0.43, 2.14)
KAI	0.9 (0.69, 1.17)	1.21 (0.56, 2.67)	0.97 (0.62, 1.53)	0.82 (0.45, 1.49)	0.79 (0.59, 1.04)	0.99 (0.6, 1.63)	0.85 (0.39, 1.83)	KAI	0.78 (0.59, 1.05)	0.92 (0.63, 1.34)	0.43 (0.35, 0.53)	0.75 (0.49, 1.16)	0.44 (0.2, 0.98)	1.11 (0.74, 1.66)	0.91 (0.66, 1.26)	0.79 (0.56, 1.13)	0.81 (0.56, 1.19)
KLTI	1.15 (0.89, 1.48)	1.54 (0.72, 3.41)	1.23 (0.8, 1.93)	1.04 (0.58, 1.89)	1 (0.77, 1.3)	1.26 (0.78, 2.07)	1.08 (0.51, 2.33)	1.28 (0.96, 1.71)	KLTI	1.17 (0.81, 1.69)	0.55 (0.45, 0.66)	0.96 (0.64, 1.46)	0.57 (0.26, 1.24)	1.42 (0.96, 2.1)	1.17 (0.86, 1.59)	1.01 (0.73, 1.42)	1.04 (0.72, 1.5)
OOMI	0.98 (0.69, 1.39)	1.32 (0.59, 3.01)	1.06 (0.64, 1.75)	0.89 (0.47, 1.69)	0.86 (0.6, 1.22)	1.08 (0.63, 1.87)	0.92 (0.41, 2.05)	1.09 (0.75, 1.59)	0.86 (0.59, 1.23)	OOMI	0.47 (0.34, 0.64)	0.82 (0.51, 1.33)	0.48 (0.21, 1.1)	1.21 (0.77, 1.92)	1 (0.67, 1.48)	0.87 (0.57, 1.31)	0.89 (0.57, 1.37)
WM	2.09 (1.77, 2.47)	2.81 (1.35, 6.04)	2.25 (1.51, 3.37)	1.89 (1.08, 3.33)	1.82 (1.52, 2.19)	2.29 (1.47, 3.62)	1.97 (0.95, 4.14)	2.32 (1.88, 2.89)	1.82 (1.51, 2.21)	2.13 (1.57, 2.91)	WM	1.75 (1.22, 2.53)	1.03 (0.49, 2.21)	2.58 (1.85, 3.64)	2.12 (1.67, 2.71)	1.85 (1.4, 2.44)	1.89 (1.39, 2.58)
SFI	1.19 (0.8, 1.78)	1.61 (0.71, 3.76)	1.28 (0.75, 2.21)	1.08 (0.55, 2.12)	1.04 (0.69, 1.56)	1.31 (0.73, 2.35)	1.12 (0.49, 2.57)	1.33 (0.86, 2.03)	1.04 (0.69, 1.57)	1.22 (0.75, 1.97)	0.57 (0.39, 0.82)	SFI	0.59 (0.26, 1.36)	1.47 (0.9, 2.43)	1.21 (0.78, 1.88)	1.05 (0.67, 1.67)	1.08 (0.67, 1.74)
SI	2.03 (0.93, 4.42)	2.74 (0.95, 7.98)	2.19 (0.92, 5.1)	1.84 (0.71, 4.72)	1.77 (0.81, 3.85)	2.23 (0.92, 5.37)	1.91 (0.66, 5.5)	2.26 (1.02, 4.96)	1.77 (0.81, 3.84)	2.07 (0.91, 4.67)	0.97 (0.45, 2.06)	1.7 (0.73, 3.92)	SI	2.51 (1.09, 5.73)	2.06 (0.92, 4.56)	1.8 (0.8, 4)	1.83 (0.81, 4.16)
SMI	0.81 (0.55, 1.18)	1.09 (0.48, 2.51)	0.87 (0.52, 1.48)	0.73 (0.38, 1.41)	0.71 (0.48, 1.04)	0.89 (0.51, 1.57)	0.76 (0.34, 1.72)	0.9 (0.6, 1.34)	0.71 (0.48, 1.04)	0.83 (0.52, 1.3)	0.39 (0.28, 0.54)	0.68 (0.41, 1.12)	0.4 (0.17, 0.92)	SMI	0.82 (0.54, 1.25)	0.72 (0.46, 1.11)	0.73 (0.46, 1.16)
SQFZI	0.98 (0.73, 1.31)	1.33 (0.61, 2.95)	1.06 (0.66, 1.7)	0.89 (0.48, 1.65)	0.86 (0.63, 1.16)	1.08 (0.65, 1.8)	0.93 (0.43, 2.02)	1.09 (0.79, 1.51)	0.86 (0.63, 1.17)	1 (0.68, 1.49)	0.47 (0.37, 0.6)	0.83 (0.53, 1.29)	0.48 (0.22, 1.09)	1.22 (0.8, 1.85)	SQFZI	0.87 (0.6, 1.25)	0.89 (0.6, 1.32)
XAPI	1.13 (0.82, 1.56)	1.52 (0.69, 3.4)	1.22 (0.75, 1.99)	1.03 (0.55, 1.92)	0.99 (0.71, 1.37)	1.24 (0.73, 2.13)	1.06 (0.49, 2.35)	1.26 (0.89, 1.79)	0.99 (0.7, 1.38)	1.15 (0.76, 1.75)	0.54 (0.41, 0.71)	0.95 (0.6, 1.5)	0.56 (0.25, 1.25)	1.4 (0.9, 2.17)	1.15 (0.8, 1.67)	XAPI	1.02 (0.68, 1.55)
XGDTI	1.11 (0.78, 1.57)	1.49 (0.67, 3.38)	1.19 (0.72, 1.98)	1 (0.53, 1.91)	0.97 (0.67, 1.38)	1.21 (0.7, 2.11)	1.04 (0.47, 2.33)	1.23 (0.84, 1.79)	0.96 (0.67, 1.39)	1.13 (0.73, 1.74)	0.53 (0.39, 0.72)	0.93 (0.58, 1.5)	0.55 (0.24, 1.24)	1.37 (0.86, 2.16)	1.12 (0.76, 1.67)	0.98 (0.65, 1.48)	XGDTI

Table S2 Results of the network meta-analysis of Survival Quality Score.

.

	ADI	CSI	DCI	DLSI	FFKSI	HCSI	HQI	KAI	KLTI	OOMI	WM	SFI	SI	SMI	SQFZI	XAPI	XGDTI
ADI	ADI	0.8 (0.44, 1.48)	1.49 (0.95, 2.36)	1.06 (0.66, 1.71)	0.96 (0.75, 1.22)	0.98 (0.45, 2.21)	2 (0.56, 8.59)	1.18 (0.87, 1.61)	0.88 (0.67, 1.15)	0.94 (0.64, 1.37)	0.34 (0.29, 0.4)	0.89 (0.59, 1.35)	1.24 (0.77, 2.03)	1.1 (0.74, 1.66)	1.04 (0.79, 1.37)	1.14 (0.81, 1.62)	1.07 (0.79, 1.45)
CSI	1.24 (0.67, 2.26)	CSI	1.86 (0.9, 3.81)	1.31 (0.63, 2.74)	1.19 (0.64, 2.19)	1.22 (0.46, 3.27)	2.49 (0.62, 11.89)	1.47 (0.77, 2.77)	1.09 (0.58, 2.02)	1.16 (0.59, 2.28)	0.42 (0.23, 0.75)	1.11 (0.55, 2.21)	1.55 (0.73, 3.24)	1.37 (0.68, 2.73)	1.29 (0.68, 2.4)	1.42 (0.73, 2.74)	1.33 (0.69, 2.5)
DCI	0.67 (0.42, 1.05)	0.54 (0.26, 1.11)	DCI	0.71 (0.38, 1.32)	0.64 (0.4, 1.02)	0.66 (0.27, 1.62)	1.34 (0.35, 6.06)	0.79 (0.48, 1.3)	0.59 (0.36, 0.94)	0.63 (0.36, 1.09)	0.23 (0.15, 0.35)	0.6 (0.34, 1.06)	0.83 (0.44, 1.56)	0.74 (0.42, 1.3)	0.7 (0.43, 1.12)	0.76 (0.45, 1.29)	0.72 (0.43, 1.18)
DLSI	0.95 (0.58, 1.52)	0.76 (0.37, 1.6)	1.41 (0.76, 2.63)	DLSI	0.91 (0.55, 1.48)	0.93 (0.38, 2.32)	1.9 (0.5, 8.61)	1.12 (0.66, 1.88)	0.83 (0.5, 1.37)	0.89 (0.5, 1.57)	0.32 (0.2, 0.51)	0.84 (0.46, 1.52)	1.18 (0.62, 2.25)	1.05 (0.58, 1.88)	0.98 (0.59, 1.63)	1.08 (0.62, 1.87)	1.01 (0.6, 1.7)
FFKSI	1.04 (0.82, 1.33)	0.84 (0.46, 1.56)	1.55 (0.98, 2.49)	1.1 (0.68, 1.81)	FFKSI	1.02 (0.46, 2.32)	2.09 (0.58, 8.98)	1.23 (0.89, 1.7)	0.92 (0.69, 1.22)	0.98 (0.66, 1.45)	0.36 (0.29, 0.43)	0.93 (0.6, 1.43)	1.3 (0.79, 2.15)	1.15 (0.76, 1.75)	1.08 (0.81, 1.46)	1.19 (0.83, 1.72)	1.11 (0.81, 1.54)
HCSI	1.02 (0.45, 2.23)	0.82 (0.31, 2.16)	1.52 (0.62, 3.67)	1.08 (0.43, 2.64)	0.98 (0.43, 2.16)	HCSI	2.04 (0.46, 10.49)	1.2 (0.52, 2.71)	0.89 (0.39, 1.99)	0.95 (0.4, 2.22)	0.35 (0.16, 0.75)	0.91 (0.38, 2.15)	1.27 (0.5, 3.12)	1.12 (0.47, 2.66)	1.06 (0.46, 2.36)	1.16 (0.49, 2.66)	1.09 (0.47, 2.46)

HQI	0.5 (0.12, 1.78)	0.4 (0.08, 1.62)	0.74 (0.16, 2.82)	0.53 (0.12, 2.01)	0.48 (0.11, 1.72)	0.49 (0.1, 2.19)	HQI	0.59 (0.14, 2.14)	0.44 (0.1, 1.58)	0.47 (0.11, 1.73)	0.17 (0.04, 0.6)	0.44 (0.1, 1.67)	0.62 (0.14, 2.39)	0.55 (0.12, 2.06)	0.52 (0.12, 1.87)	0.57 (0.13, 2.08)	0.53 (0.12, 1.94)
KAI	0.85 (0.62, 1.15)	0.68 (0.36, 1.31)	1.27 (0.77, 2.1)	0.9 (0.53, 1.52)	0.81 (0.59, 1.12)	0.83 (0.37, 1.93)	1.7 (0.47, 7.39)	KAI	0.74 (0.53, 1.05)	0.79 (0.51, 1.23)	0.29 (0.22, 0.38)	0.76 (0.47, 1.21)	1.05 (0.62, 1.8)	0.94 (0.59, 1.49)	0.88 (0.62, 1.25)	0.97 (0.64, 1.46)	0.91 (0.62, 1.32)
KLTI	1.14 (0.87, 1.48)	0.92 (0.49, 1.72)	1.7 (1.06, 2.76)	1.2 (0.73, 1.99)	1.09 (0.82, 1.45)	1.12 (0.5, 2.55)	2.28 (0.63, 9.84)	1.34 (0.95, 1.9)	KLTI	1.07 (0.71, 1.61)	0.39 (0.31, 0.48)	1.01 (0.65, 1.58)	1.41 (0.86, 2.37)	1.26 (0.82, 1.94)	1.18 (0.86, 1.62)	1.3 (0.89, 1.9)	1.22 (0.87, 1.71)
OOMI	1.07 (0.73, 1.56)	0.86 (0.44, 1.71)	1.59 (0.92, 2.77)	1.13 (0.64, 2)	1.03 (0.69, 1.52)	1.05 (0.45, 2.5)	2.14 (0.58, 9.52)	1.26 (0.81, 1.95)	0.94 (0.62, 1.42)	OOMI	0.36 (0.26, 0.52)	0.95 (0.57, 1.6)	1.33 (0.75, 2.38)	1.18 (0.71, 1.97)	1.11 (0.73, 1.69)	1.22 (0.76, 1.95)	1.14 (0.74, 1.77)
WM	2.93 (2.52, 3.42)	2.36 (1.33, 4.27)	4.37 (2.87, 6.75)	3.1 (1.98, 4.9)	2.81 (2.33, 3.4)	2.88 (1.33, 6.38)	5.87 (1.67, 24.97)	3.46 (2.66, 4.52)	2.57 (2.08, 3.2)	2.74 (1.94, 3.9)	WM	2.61 (1.78, 3.85)	3.64 (2.32, 5.82)	3.24 (2.24, 4.72)	3.05 (2.42, 3.83)	3.34 (2.46, 4.59)	3.13 (2.41, 4.09)
SFI	1.12 (0.74, 1.7)	0.9 (0.45, 1.83)	1.67 (0.94, 2.98)	1.19 (0.66, 2.16)	1.08 (0.7, 1.65)	1.1 (0.47, 2.67)	2.25 (0.6, 10.04)	1.32 (0.83, 2.11)	0.99 (0.63, 1.53)	1.05 (0.62, 1.77)	0.38 (0.26, 0.56)	SFI	1.39 (0.77, 2.56)	1.24 (0.73, 2.12)	1.17 (0.74, 1.82)	1.28 (0.78, 2.1)	1.2 (0.75, 1.92)
SI	0.8 (0.49, 1.3)	0.65 (0.31, 1.37)	1.2 (0.64, 2.25)	0.85 (0.44, 1.62)	0.77 (0.47, 1.26)	0.79 (0.32, 1.99)	1.61 (0.42, 7.35)	0.95 (0.56, 1.61)	0.71 (0.42, 1.17)	0.75 (0.42, 1.34)	0.27 (0.17, 0.43)	0.72 (0.39, 1.3)	SI	0.89 (0.49, 1.6)	0.84 (0.5, 1.39)	0.92 (0.52, 1.6)	0.86 (0.5, 1.45)
SMI	0.91 (0.6, 1.35)	0.73 (0.37, 1.47)	1.35 (0.77, 2.38)	0.96 (0.53, 1.72)	0.87 (0.57, 1.31)	0.89 (0.38, 2.14)	1.81 (0.49, 8.07)	1.07 (0.67, 1.68)	0.8 (0.52, 1.22)	0.85 (0.51, 1.41)	0.31 (0.21, 0.45)	0.81 (0.47, 1.38)	1.13 (0.62, 2.04)	SMI	0.94 (0.61, 1.45)	1.03 (0.64, 1.68)	0.97 (0.61, 1.52)
SQFZI	0.96 (0.73, 1.27)	0.77 (0.42, 1.46)	1.44 (0.89, 2.34)	1.02 (0.62, 1.7)	0.92 (0.69, 1.24)	0.95 (0.42, 2.17)	1.93 (0.54, 8.35)	1.13 (0.8, 1.61)	0.85 (0.62, 1.16)	0.9 (0.59, 1.37)	0.33 (0.26, 0.41)	0.86 (0.55, 1.34)	1.2 (0.72, 2.02)	1.06 (0.69, 1.65)	SQFZI	1.1 (0.75, 1.62)	1.03 (0.73, 1.46)
XAPI	0.88 (0.62, 1.24)	0.7 (0.37, 1.38)	1.31 (0.77, 2.23)	0.93 (0.54, 1.61)	0.84 (0.58, 1.21)	0.86 (0.38, 2.02)	1.76 (0.48, 7.71)	1.03 (0.69, 1.56)	0.77 (0.53, 1.12)	0.82 (0.51, 1.31)	0.3 (0.22, 0.41)	0.78 (0.48, 1.28)	1.09 (0.63, 1.91)	0.97 (0.6, 1.57)	0.91 (0.62, 1.34)	XAPI	0.94 (0.62, 1.41)
XGDTI	0.94 (0.69, 1.27)	0.75 (0.4, 1.44)	1.4 (0.85, 2.31)	0.99 (0.59, 1.67)	0.9 (0.65, 1.24)	0.92 (0.41, 2.12)	1.87 (0.52, 8.17)	1.1 (0.76, 1.61)	0.82 (0.58, 1.16)	0.88 (0.56, 1.36)	0.32 (0.24, 0.41)	0.83 (0.52, 1.33)	1.16 (0.69, 1.99)	1.03 (0.66, 1.64)	0.97 (0.68, 1.38)	1.07 (0.71, 1.61)	XGDTI

Table S3 Results of the network meta-analysis of Incidence of GI Adverse Reactions.

	ADI	CSI	DCI	DLSI	FFKSI	HCSI	HQI	KAI	KLTI	OOMI	WM	SFI	SI	SMI	SQFZI	XAPI	XGDTI
ADI	ADI	0.43 (0.07, 2.18)	0.48 (0.27, 0.87)	0.61 (0.32, 1.16)	0.69 (0.49, 0.95)	0.6 (0.29, 1.18)	2.02 (0.49, 8.26)	0.82 (0.57, 1.18)	0.93 (0.66, 1.33)	1.97 (1.3, 2.97)	2.15 (1.75, 2.65)	0.93 (0.55, 1.56)	1.28 (0.64, 2.52)	0.72 (0.44, 1.18)	0.65 (0.44, 0.93)	1.17 (0.69, 1.98)	0.8 (0.51, 1.24)
CSI	2.34 (0.46, 15.04)	CSI	1.13 (0.2, 7.85)	1.43 (0.25, 9.96)	1.6 (0.31, 10.38)	1.39 (0.24, 9.82)	4.77 (0.55, 47.73)	1.92 (0.37, 12.48)	2.19 (0.42, 14.18)	4.61 (0.88, 30.33)	5.04 (1, 32.1)	2.18 (0.4, 14.62)	2.99 (0.52, 21.14)	1.68 (0.31, 11.27)	1.51 (0.29, 9.84)	2.73 (0.5, 18.45)	1.87 (0.36, 12.31)
DCI	2.06 (1.15, 3.76)	0.88 (0.13, 4.9)	DCI	1.26 (0.55, 2.89)	1.41 (0.77, 2.62)	1.23 (0.52, 2.9)	4.17 (0.93, 18.78)	1.69 (0.9, 3.21)	1.93 (1.04, 3.62)	4.06 (2.11, 7.93)	4.44 (2.58, 7.81)	1.92 (0.93, 4.01)	2.64 (1.12, 6.22)	1.48 (0.73, 3.06)	1.33 (0.71, 2.53)	2.41 (1.16, 5.09)	1.65 (0.84, 3.27)
DLSI	1.64 (0.86, 3.14)	0.7 (0.1, 3.96)	0.79 (0.35, 1.82)	DLSI	1.12 (0.58, 2.19)	0.97 (0.39, 2.39)	3.31 (0.72, 15.18)	1.34 (0.68, 2.68)	1.53 (0.78, 3.03)	3.23 (1.59, 6.59)	3.53 (1.92, 6.55)	1.52 (0.7, 3.34)	2.09 (0.85, 5.14)	1.18 (0.55, 2.53)	1.06 (0.53, 2.11)	1.91 (0.87, 4.2)	1.31 (0.64, 2.72)
FFKSI	1.46 (1.05, 2.03)	0.62 (0.1, 3.21)	0.71 (0.38, 1.3)	0.89 (0.46, 1.73)	FFKSI	0.87 (0.42, 1.75)	2.95 (0.71, 12.15)	1.2 (0.8, 1.78)	1.36 (0.93, 2.01)	2.88 (1.86, 4.47)	3.14 (2.44, 4.07)	1.36 (0.79, 2.34)	1.87 (0.92, 3.74)	1.05 (0.62, 1.77)	0.94 (0.63, 1.41)	1.7 (0.98, 2.96)	1.17 (0.73, 1.86)
HCSI	1.68 (0.85, 3.39)	0.72 (0.1, 4.15)	0.81 (0.35, 1.94)	1.03 (0.42, 2.54)	1.15 (0.57, 2.35)	HCSI	3.39 (0.73, 15.97)	1.38 (0.67, 2.87)	1.57 (0.77, 3.26)	3.31 (1.57, 7.08)	3.62 (1.89, 7.09)	1.56 (0.69, 3.56)	2.15 (0.85, 5.44)	1.21 (0.55, 2.71)	1.08 (0.53, 2.27)	1.96 (0.87, 4.51)	1.34 (0.63, 2.92)
HQI	0.5 (0.12, 2.04)	0.21 (0.02, 1.83)	0.24 (0.05, 1.07)	0.3 (0.07, 1.39)	0.34 (0.08, 1.4)	0.29 (0.06, 1.38)	HQI	0.41 (0.1, 1.69)	0.46 (0.11, 1.92)	0.98 (0.23, 4.12)	1.07 (0.27, 4.31)	0.46 (0.11, 2.02)	0.63 (0.14, 2.95)	0.36 (0.08, 1.54)	0.32 (0.08, 1.34)	0.58 (0.13, 2.53)	0.4 (0.09, 1.68)
KAI	1.22 (0.85, 1.77)	0.52 (0.08, 2.71)	0.59 (0.31, 1.11)	0.75 (0.37, 1.48)	0.84 (0.56, 1.24)	0.73 (0.35, 1.49)	2.47 (0.59, 10.25)	KAI	1.14 (0.75, 1.74)	2.41 (1.5, 3.85)	2.63 (1.94, 3.57)	1.13 (0.64, 2)	1.56 (0.75, 3.19)	0.88 (0.51, 1.51)	0.79 (0.51, 1.21)	1.43 (0.8, 2.53)	0.98 (0.6, 1.6)
KLTI	1.07 (0.75, 1.52)	0.46 (0.07, 2.36)	0.52 (0.28, 0.96)	0.65 (0.33, 1.28)	0.73 (0.5, 1.07)	0.64 (0.31, 1.29)	2.16 (0.52, 8.97)	0.88 (0.58, 1.33)	KLTI	2.11 (1.33, 3.33)	2.3 (1.73, 3.07)	0.99 (0.57, 1.74)	1.37 (0.67, 2.77)	0.77 (0.45, 1.31)	0.69 (0.45, 1.05)	1.25 (0.71, 2.2)	0.86 (0.53, 1.38)
OOMI	0.51 (0.34, 0.77)	0.22 (0.03, 1.14)	0.25 (0.13, 0.47)	0.31 (0.15, 0.63)	0.35 (0.22, 0.54)	0.3 (0.14, 0.64)	1.02 (0.24, 4.32)	0.42 (0.26, 0.67)	0.47 (0.3, 0.75)	OOMI	1.09 (0.77, 1.56)	0.47 (0.26, 0.86)	0.65 (0.31, 1.36)	0.36 (0.2, 0.65)	0.33 (0.2, 0.52)	0.59 (0.32, 1.09)	0.41 (0.24, 0.69)
WM	0.46 (0.38, 0.57)	0.2 (0.03, 0.90)	0.23 (0.13, 0.39)	0.28 (0.15, 0.52)	0.32 (0.25, 0.41)	0.28 (0.14, 0.53)	0.94 (0.23, 3.77)	0.38 (0.28, 0.52)	0.43 (0.33, 0.58)	0.91 (0.64, 1.31)	WM	0.43 (0.27, 0.7)	0.59 (0.31, 1.13)	0.33 (0.21, 0.52)	0.3 (0.22, 0.41)	0.54 (0.33, 0.88)	0.37 (0.25, 0.55)
SFI	1.08 (0.64, 1.83)	0.46 (0.07, 2.5)	0.52 (0.25, 1.08)	0.66 (0.3, 1.43)	0.74 (0.43, 1.27)	0.64 (0.28, 1.44)	2.17 (0.5, 9.49)	0.88 (0.5, 1.56)	1.01 (0.58, 1.77)	2.12 (1.16, 3.87)	2.32 (1.44, 3.77)	SFI	1.37 (0.61, 3.08)	0.77 (0.4, 1.5)	0.69 (0.39, 1.23)	1.26 (0.63, 2.5)	0.86 (0.46, 1.6)
SI	0.78 (0.4, 1.56)	0.33 (0.05, 1.92)	0.38 (0.16, 0.89)	0.48 (0.19, 1.17)	0.54 (0.27, 1.09)	0.47 (0.18, 1.17)	1.58 (0.34, 7.36)	0.64 (0.31, 1.33)	0.73 (0.36, 1.5)	1.54 (0.74, 3.26)	1.69 (0.88, 3.26)	0.73 (0.32, 1.64)	SI	0.56 (0.25, 1.25)	0.51 (0.25, 1.04)	0.91 (0.41, 2.08)	0.63 (0.29, 1.35)
SMI	1.39 (0.85, 2.3)	0.59 (0.09, 3.2)	0.67 (0.33, 1.38)	0.85 (0.39, 1.82)	0.95 (0.57, 1.6)	0.83 (0.37, 1.83)	2.81 (0.65, 12.22)	1.14 (0.66, 1.98)	1.3 (0.76, 2.23)	2.74 (1.54, 4.89)	3 (1.91, 4.74)	1.29 (0.67, 2.51)	1.78 (0.8, 3.93)	SMI	0.9 (0.52, 1.56)	1.63 (0.83, 3.17)	1.11 (0.61, 2.03)
SQFZI	1.55 (1.07, 2.25)	0.66 (0.1, 3.43)	0.75 (0.4, 1.41)	0.95 (0.47, 1.88)	1.06 (0.71, 1.58)	0.92 (0.44, 1.89)	3.13 (0.75, 13.04)	1.27 (0.82, 1.96)	1.45 (0.95, 2.21)	3.06 (1.9, 4.89)	3.34 (2.46, 4.55)	1.44 (0.81, 2.55)	1.98 (0.96, 4.05)	1.11 (0.64, 1.92)	SQFZI	1.81 (1.02, 3.22)	1.24 (0.75, 2.04)
XAPI	0.86 (0.5, 1.46)	0.37 (0.05, 2)	0.42 (0.2, 0.86)	0.52 (0.24, 1.14)	0.59 (0.34, 1.02)	0.51 (0.22, 1.15)	1.73 (0.4, 7.58)	0.7 (0.39, 1.25)	0.8 (0.46, 1.42)	1.69 (0.92, 3.09)	1.85 (1.13, 3.01)	0.79 (0.4, 1.58)	1.09 (0.48, 2.46)	0.62 (0.32, 1.2)	0.55 (0.31, 0.98)	XAPI	0.68 (0.37, 1.28)
XGDTI	1.25 (0.81, 1.94)	0.54 (0.08, 2.82)	0.61 (0.31, 1.19)	0.76 (0.37, 1.57)	0.86 (0.54, 1.36)	0.74 (0.34, 1.59)	2.52 (0.59, 10.71)	1.03 (0.62, 1.68)	1.17 (0.72, 1.9)	2.47 (1.45, 4.17)	2.69 (1.83, 3.98)	1.16 (0.63, 2.16)	1.6 (0.74, 3.4)	0.9 (0.49, 1.64)	0.81 (0.49, 1.33)	1.46 (0.78, 2.72)	XGDTI

Table S4 Results of the network meta-analysis of incidence of Leukopenia.

.

	ADI	CSI	DCI	DLSI	FFKSI	HCSI	HQI	KAI	KLTI	OOMI	WM	SFI	SI	SMI	SQFZI	XAPI	XGDTI
ADI	ADI	1.39 (0.13, 11.14)	0.96 (0.57, 1.62)	0.88 (0.32, 2.28)	0.92 (0.65, 1.3)	1.36 (0.76, 2.38)	2.06 (0.96, 4.47)	0.93 (0.62, 1.4)	1.13 (0.77, 1.67)	1.03 (0.66, 1.61)	2.87 (2.31, 3.61)	1.36 (0.77, 2.4)	1.22 (0.73, 2.02)	0.82 (0.49, 1.34)	0.91 (0.62, 1.33)	0.95 (0.59, 1.54)	1.18 (0.78, 1.78)
CSI	0.72 (0.09, 7.5)	CSI	0.69 (0.08, 7.53)	0.63 (0.06, 7.64)	0.66 (0.08, 6.9)	0.98 (0.12, 10.53)	1.49 (0.17, 16.9)	0.67 (0.08, 7)	0.81 (0.1, 8.55)	0.74 (0.09, 7.91)	2.06 (0.26, 21.26)	0.98 (0.12, 10.68)	0.87 (0.11, 9.46)	0.59 (0.07, 6.3)	0.65 (0.08, 6.84)	0.68 (0.08, 7.32)	0.85 (0.1, 8.94)
DCI	1.04 (0.62, 1.75)	1.45 (0.13, 12.09)	DCI	0.91 (0.31, 2.6)	0.95 (0.56, 1.64)	1.42 (0.7, 2.85)	2.15 (0.9, 5.14)	0.97 (0.54, 1.74)	1.18 (0.67, 2.09)	1.08 (0.59, 1.98)	2.99 (1.88, 4.82)	1.42 (0.7, 2.86)	1.27 (0.66, 2.44)	0.85 (0.44, 1.63)	0.94 (0.54, 1.66)	0.99 (0.52, 1.87)	1.22 (0.68, 2.21)
DLSI	1.14 (0.44, 3.12)	1.59 (0.13, 15.69)	1.1 (0.38, 3.24)	DLSI	1.05 (0.4, 2.89)	1.55 (0.53, 4.69)	2.36 (0.72, 8.13)	1.06 (0.39, 3)	1.29 (0.48, 3.64)	1.18 (0.43, 3.4)	3.28 (1.29, 8.79)	1.56 (0.54, 4.72)	1.39 (0.49, 4.13)	0.93 (0.33, 2.74)	1.04 (0.39, 2.9)	1.08 (0.39, 3.17)	1.34 (0.5, 3.81)
FFKSI	1.09 (0.77, 1.54)	1.52 (0.14, 12.22)	1.05 (0.61, 1.79)	0.96 (0.35, 2.52)	FFKSI	1.48 (0.82, 2.66)	2.25 (1.03, 4.92)	1.01 (0.66, 1.56)	1.23 (0.82, 1.87)	1.13 (0.71, 1.8)	3.13 (2.41, 4.1)	1.49 (0.83, 2.67)	1.33 (0.78, 2.25)	0.89 (0.52, 1.5)	0.99 (0.66, 1.49)	1.04 (0.63, 1.72)	1.28 (0.83, 2)
HCSI	0.74 (0.42, 1.31)	1.02 (0.09, 8.63)	0.71 (0.35, 1.43)	0.64 (0.21, 1.88)	0.67 (0.38, 1.23)	HCSI	1.52 (0.62, 3.77)	0.68 (0.37, 1.29)	0.83 (0.45, 1.56)	0.76 (0.4, 1.48)	2.11 (1.26, 3.63)	1 (0.48, 2.12)	0.89 (0.45, 1.81)	0.6 (0.3, 1.2)	0.67 (0.37, 1.24)	0.7 (0.36, 1.39)	0.86 (0.46, 1.64)
HQI	0.48 (0.22, 1.05)	0.67 (0.06, 5.98)	0.46 (0.19, 1.12)	0.42 (0.12, 1.39)	0.44 (0.2, 0.97)	0.66 (0.27, 1.61)	HQI	0.45 (0.2, 1.01)	0.55 (0.25, 1.22)	0.5 (0.22, 1.15)	1.39 (0.67, 2.91)	0.66 (0.27, 1.62)	0.59 (0.25, 1.4)	0.39 (0.17, 0.93)	0.44 (0.2, 0.98)	0.46 (0.2, 1.07)	0.57 (0.25, 1.29)
KAI	1.08 (0.72, 1.62)	1.49 (0.14, 12.18)	1.03 (0.58, 1.84)	0.94 (0.33, 2.54)	0.99 (0.64, 1.52)	1.46 (0.77, 2.72)	2.22 (0.99, 5.01)	KAI	1.22 (0.77, 1.94)	1.11 (0.67, 1.86)	3.09 (2.2, 4.37)	1.47 (0.78, 2.73)	1.31 (0.74, 2.32)	0.88 (0.49, 1.54)	0.98 (0.62, 1.55)	1.02 (0.59, 1.77)	1.27 (0.78, 2.07)
KLTI	0.89 (0.6, 1.3)	1.23 (0.12, 9.92)	0.85 (0.48, 1.49)	0.78 (0.27, 2.06)	0.81 (0.53, 1.22)	1.21 (0.64, 2.2)	1.83 (0.82, 4.05)	0.82 (0.51, 1.31)	KLTI	0.92 (0.56, 1.51)	2.54 (1.86, 3.48)	1.21 (0.65, 2.22)	1.08 (0.61, 1.87)	0.72 (0.41, 1.24)	0.8 (0.51, 1.25)	0.84 (0.49, 1.42)	1.04 (0.65, 1.66)
OOMI	0.97 (0.62, 1.5)	1.34 (0.13, 11.02)	0.93 (0.51, 1.69)	0.85 (0.29, 2.31)	0.89 (0.55, 1.41)	1.32 (0.68, 2.5)	2 (0.87, 4.56)	0.9 (0.54, 1.5)	1.09 (0.66, 1.8)	OOMI	2.77 (1.9, 4.08)	1.32 (0.69, 2.51)	1.18 (0.64, 2.13)	0.79 (0.43, 1.42)	0.88 (0.53, 1.44)	0.92 (0.51, 1.63)	1.14 (0.68, 1.91)
WM	0.35 (0.28, 0.43)	0.48 (0.05, 3.82)	0.33 (0.21, 0.53)	0.31 (0.11, 0.77)	0.32 (0.24, 0.42)	0.47 (0.28, 0.79)	0.72 (0.34, 1.5)	0.32 (0.23, 0.45)	0.39 (0.29, 0.54)	0.36 (0.24, 0.53)	WM	0.48 (0.28, 0.8)	0.42 (0.27, 0.67)	0.28 (0.18, 0.44)	0.32 (0.23, 0.43)	0.33 (0.22, 0.5)	0.41 (0.29, 0.58)
SFI	0.73 (0.42, 1.3)	1.02 (0.09, 8.55)	0.7 (0.35, 1.43)	0.64 (0.21, 1.87)	0.67 (0.37, 1.21)	1 (0.47, 2.08)	1.51 (0.62, 3.73)	0.68 (0.37, 1.27)	0.83 (0.45, 1.54)	0.76 (0.4, 1.45)	2.1 (1.25, 3.56)	SFI	0.89 (0.44, 1.79)	0.6 (0.3, 1.19)	0.67 (0.36, 1.23)	0.7 (0.36, 1.37)	0.86 (0.46, 1.62)
SI	0.82 (0.49, 1.37)	1.14 (0.11, 9.47)	0.79 (0.41, 1.52)	0.72 (0.24, 2.04)	0.75 (0.44, 1.28)	1.12 (0.55, 2.24)	1.7 (0.71, 4.05)	0.76 (0.43, 1.35)	0.93 (0.54, 1.63)	0.85 (0.47, 1.55)	2.36 (1.5, 3.75)	1.12 (0.56, 2.25)	SI	0.67 (0.35, 1.27)	0.75 (0.43, 1.3)	0.78 (0.42, 1.46)	0.97 (0.54, 1.72)
SMI	1.23 (0.75, 2.03)	1.71 (0.16, 14.16)	1.18 (0.61, 2.26)	1.07 (0.37, 3.05)	1.12 (0.67, 1.91)	1.67 (0.83, 3.31)	2.54 (1.07, 6.02)	1.14 (0.65, 2.02)	1.39 (0.8, 2.43)	1.27 (0.7, 2.3)	3.52 (2.26, 5.57)	1.67 (0.84, 3.33)	1.49 (0.79, 2.84)	SMI	1.11 (0.65, 1.93)	1.16 (0.63, 2.18)	1.44 (0.82, 2.57)
SQFZI	1.1 (0.75, 1.61)	1.53 (0.15, 12.36)	1.06 (0.6, 1.85)	0.97 (0.34, 2.58)	1.01 (0.67, 1.52)	1.5 (0.81, 2.73)	2.28 (1.02, 5.06)	1.02 (0.65, 1.62)	1.24 (0.8, 1.94)	1.14 (0.7, 1.87)	3.16 (2.33, 4.33)	1.5 (0.81, 2.75)	1.34 (0.77, 2.32)	0.9 (0.52, 1.54)	SQFZI	1.05 (0.62, 1.77)	1.3 (0.81, 2.07)
XAPI	1.05 (0.65, 1.7)	1.47 (0.14, 12.05)	1.01 (0.53, 1.91)	0.92 (0.32, 2.57)	0.97 (0.58, 1.6)	1.43 (0.72, 2.8)	2.17 (0.93, 5.09)	0.98 (0.57, 1.69)	1.19 (0.7, 2.02)	1.09 (0.61, 1.94)	3.02 (1.98, 4.65)	1.44 (0.73, 2.8)	1.28 (0.68, 2.4)	0.86 (0.46, 1.59)	0.96 (0.57, 1.62)	XAPI	1.24 (0.71, 2.15)
XGDTI	0.85 (0.56, 1.29)	1.18 (0.11, 9.65)	0.82 (0.45, 1.47)	0.74 (0.26, 2.02)	0.78 (0.5, 1.21)	1.16 (0.61, 2.16)	1.75 (0.78, 3.96)	0.79 (0.48, 1.29)	0.96 (0.6, 1.54)	0.88 (0.52, 1.48)	2.44 (1.72, 3.47)	1.16 (0.62, 2.16)	1.03 (0.58, 1.84)	0.69 (0.39, 1.22)	0.77 (0.48, 1.23)	0.81 (0.46, 1.4)	XGDTI

Table S5 Results of the network meta-analysis of incidence of thrombocytopenia.

	ADI	DCI	DLSI	FFKSI	HCSI	KAI	KLTI	OOMI	WM	SFI	WM	SMI	SQFZI	XAPI	XGDTI
ADI	ADI	0.34 (0.13, 0.85)	0.52 (0.12, 2.05)	0.96 (0.64, 1.45)	0.41 (0.19, 0.89)	0.72 (0.4, 1.29)	0.89 (0.54, 1.46)	1.79 (0.5, 6.43)	2.19 (1.67, 2.89)	0.88 (0.48, 1.62)	2.38 (1.14, 5)	0.63 (0.37, 1.08)	1.24 (0.82, 1.88)	1.26 (0.77, 2.07)	0.81 (0.44, 1.49)
DCI	2.93 (1.17, 7.8)	DCI	1.53 (0.29, 7.99)	2.8 (1.11, 7.55)	1.21 (0.39, 3.99)	2.1 (0.76, 6.18)	2.62 (1, 7.36)	5.27 (1.15, 24.97)	6.4 (2.68, 16.47)	2.58 (0.92, 7.65)	6.98 (2.3, 22.25)	1.86 (0.69, 5.3)	3.64 (1.45, 9.82)	3.68 (1.4, 10.32)	2.38 (0.85, 7.05)
DLSI	1.9 (0.49, 8.26)	0.65 (0.13, 3.5)	DLSI	1.83 (0.47, 7.95)	0.79 (0.17, 3.94)	1.37 (0.32, 6.3)	1.71 (0.42, 7.66)	3.43 (0.54, 23.06)	4.16 (1.1, 17.68)	1.68 (0.4, 7.8)	4.54 (1.01, 22.33)	1.21 (0.29, 5.47)	2.37 (0.6, 10.39)	2.4 (0.59, 10.71)	1.54 (0.37, 7.21)
FFKSI	1.05 (0.69, 1.57)	0.36 (0.13, 0.9)	0.55 (0.13, 2.15)	FFKSI	0.43 (0.2, 0.94)	0.75 (0.41, 1.37)	0.93 (0.55, 1.56)	1.87 (0.52, 6.75)	2.29 (1.69, 3.11)	0.92 (0.49, 1.72)	2.48 (1.17, 5.24)	0.66 (0.38, 1.14)	1.3 (0.84, 2.01)	1.31 (0.78, 2.2)	0.85 (0.45, 1.57)
HCSI	2.42 (1.12, 5.23)	0.83 (0.25, 2.57)	1.27 (0.25, 5.74)	2.32 (1.07, 5.09)	HCSI	1.73 (0.71, 4.24)	2.16 (0.94, 4.97)	4.33 (1.03, 18.29)	5.29 (2.6, 10.95)	2.13 (0.86, 5.25)	5.75 (2.14, 15.59)	1.53 (0.65, 3.6)	3.01 (1.38, 6.62)	3.04 (1.33, 6.99)	1.96 (0.8, 4.84)

KAI	1.4 (0.77, 2.52)	0.48 (0.16, 1.32)	0.73 (0.16, 3.08)	1.34 (0.73, 2.47)	0.58 (0.24, 1.4)	KAI	1.25 (0.64, 2.44)	2.5 (0.64, 9.64)	3.05 (1.82, 5.18)	1.23 (0.58, 2.61)	3.32 (1.4, 7.87)	0.89 (0.44, 1.78)	1.74 (0.94, 3.2)	1.76 (0.9, 3.43)	1.13 (0.53, 2.41)
KLTI	1.12 (0.68, 1.84)	0.38 (0.14, 1)	0.59 (0.13, 2.38)	1.07 (0.64, 1.8)	0.46 (0.2, 1.06)	0.8 (0.41, 1.56)	KLTI	2 (0.54, 7.51)	2.44 (1.62, 3.73)	0.98 (0.5, 1.96)	2.65 (1.2, 5.94)	0.71 (0.38, 1.32)	1.39 (0.83, 2.35)	1.41 (0.79, 2.54)	0.91 (0.46, 1.8)
OOMI	0.56 (0.16, 2)	0.19 (0.04, 0.87)	0.29 (0.04, 1.84)	0.54 (0.15, 1.94)	0.23 (0.05, 0.97)	0.4 (0.1, 1.55)	0.5 (0.13, 1.86)	OOMI	1.22 (0.35, 4.27)	0.49 (0.13, 1.93)	1.33 (0.32, 5.49)	0.35 (0.09, 1.34)	0.7 (0.19, 2.52)	0.7 (0.19, 2.63)	0.45 (0.12, 1.77)
WM	0.46 (0.35, 0.6)	0.16 (0.06, 0.37)	0.24 (0.06, 0.91)	0.44 (0.32, 0.59)	0.19 (0.09, 0.38)	0.33 (0.19, 0.55)	0.41 (0.27, 0.62)	0.82 (0.23, 2.84)	WM	0.4 (0.23, 0.69)	1.09 (0.55, 2.16)	0.29 (0.18, 0.46)	0.57 (0.41, 0.78)	0.58 (0.38, 0.87)	0.37 (0.21, 0.64)
SFI	1.14 (0.62, 2.09)	0.39 (0.13, 1.09)	0.6 (0.13, 2.53)	1.09 (0.58, 2.04)	0.47 (0.19, 1.16)	0.81 (0.38, 1.74)	1.02 (0.51, 2.01)	2.04 (0.52, 7.91)	2.49 (1.44, 4.31)	SFI	2.7 (1.13, 6.52)	0.72 (0.35, 1.47)	1.42 (0.75, 2.66)	1.43 (0.72, 2.84)	0.92 (0.43, 1.99)
SI	0.42 (0.2, 0.88)	0.14 (0.04, 0.43)	0.22 (0.04, 0.99)	0.4 (0.19, 0.85)	0.17 (0.06, 0.47)	0.3 (0.13, 0.72)	0.38 (0.17, 0.83)	0.75 (0.18, 3.13)	0.92 (0.46, 1.83)	0.37 (0.15, 0.89)	SI	0.27 (0.12, 0.61)	0.52 (0.25, 1.11)	0.53 (0.24, 1.18)	0.34 (0.14, 0.82)
SMI	1.58 (0.93, 2.69)	0.54 (0.19, 1.44)	0.83 (0.18, 3.39)	1.51 (0.88, 2.63)	0.65 (0.28, 1.53)	1.13 (0.56, 2.26)	1.41 (0.76, 2.62)	2.82 (0.75, 10.72)	3.44 (2.19, 5.49)	1.39 (0.68, 2.83)	3.74 (1.65, 8.59)	SMI	1.96 (1.13, 3.43)	1.98 (1.08, 3.7)	1.28 (0.63, 2.61)
SQFZI	0.8 (0.53, 1.21)	0.27 (0.1, 0.69)	0.42 (0.1, 1.67)	0.77 (0.5, 1.2)	0.33 (0.15, 0.73)	0.58 (0.31, 1.06)	0.72 (0.43, 1.2)	1.44 (0.4, 5.22)	1.76 (1.29, 2.42)	0.71 (0.38, 1.33)	1.91 (0.9, 4.07)	0.51 (0.29, 0.89)	SQFZI	1.01 (0.6, 1.71)	0.65 (0.35, 1.23)
XAPI	0.8 (0.48, 1.3)	0.27 (0.1, 0.71)	0.42 (0.09, 1.69)	0.76 (0.45, 1.27)	0.33 (0.14, 0.75)	0.57 (0.29, 1.11)	0.71 (0.39, 1.27)	1.42 (0.38, 5.31)	1.74 (1.15, 2.63)	0.7 (0.35, 1.38)	1.89 (0.85, 4.2)	0.5 (0.27, 0.93)	0.99 (0.59, 1.66)	XAPI	0.64 (0.32, 1.28)
XGDTI	1.23 (0.67, 2.27)	0.42 (0.14, 1.18)	0.65 (0.14, 2.73)	1.18 (0.64, 2.21)	0.51 (0.21, 1.25)	0.88 (0.41, 1.88)	1.1 (0.55, 2.18)	2.2 (0.56, 8.65)	2.7 (1.57, 4.69)	1.08 (0.5, 2.35)	2.93 (1.22, 7.08)	0.78 (0.38, 1.59)	1.54 (0.82, 2.88)	1.55 (0.78, 3.09)	XGDTI
